# Feasibility of Supplying Ruminally Protected Lysine and Methionine to Periparturient Dairy Cows on the Efficiency of Subsequent Lactation

**DOI:** 10.3389/fvets.2022.892709

**Published:** 2022-06-14

**Authors:** Samy A. Elsaadawy, Zhaohai Wu, Dengpan Bu

**Affiliations:** ^1^State Key Laboratory of Animal Nutrition, Institute of Animal Sciences, Chinese Academy of Agricultural Sciences, Beijing, China; ^2^Joint Laboratory on Integrated Crop-Tree-Livestock Systems of the Chinese Academy of Agricultural Sciences (CAAS), Ethiopian Institute of Agricultural Research (EIAR) and World Agroforestry Centre (ICRAF), Beijing, China; ^3^Hunan Co-Innovation Center of Safety Animal Production, Changsha, China

**Keywords:** amino acids, peak of milk, metabolizable protein, pregnancy rate, β-hydroxybutyrate, energy balance, milk production, body condition score

## Abstract

The objective of this study was to evaluate the effects of supplying ruminally protected Lys (RPL) and ruminally protected Met (RPM) to transition cows' diets on the efficiency of subsequent lactation. A total of 120 prepartum Holstein cows were assigned into four treatments blocked by the anticipated calving date, previous lactation milk yield, number of lactations, and body condition score and fed either RPL, RPM, or the combination (RPML) or control diet (CON) throughout the transition period (3 weeks before till 3 weeks after calving). From 22 to 150 days in milk (DIM), all animals (100 cows) were fed a combination of RPM and RPL (0.17% RPM and 0.41% RPL of DM; *n* = 25 cows/treatment) as follows; CON–RPML, RPM–RPML, RPL–RPML, and RPML–RPML. Milk production and dry matter intake (DMI) were measured daily; milk and blood samples were taken at 21, 30, 60, 90, 120, and 150 DIM. Supplemented amino acids (AA) were mixed with the premix and added to the total mixed ration during the experiment. DMI (*p* < 0.001) and energy-corrected milk (ECM, *p* = 0.04) were higher for cows that were fed RPML–RPML than other cows. Compared with CON–RPML, yields of milk total protein, lactose, and nitrogen efficiency were increased (*p* < 0.01), whereas milk urea nitrogen (MUN; *p* = 0.002) was decreased for other treatments. However, supplemental AA did not affect milk lactose percentage, fat yield, feed efficiency, or serum total protein concentration (*p* > 0.10). Transition cows that consumed AA had a greater peak of milk yield (*p* < 0.01), as well as quickly reached the peak of milk (*p* < 0.004). There were differences in β-hydroxybutyrate concentration during the early lactation, with a lower level for AA groups (*p* < 0.05), and the difference faded with the progression of lactation (*p* > 0.10). Fertility efficiency as measured by pregnancy rate was improved by supplemental AA during the perinatal period (*p* < 0.05). In conclusion, transition cows consumed RPM and RPL, increased post-calving DMI, milk production, milk protein yield, nitrogen efficiency, and improved fertility performance.

## Introduction

Balanced nutrition is necessary for the body to perform its various functions optimally while maintaining life. Nutrition deficiency causes a reduction of fertility (1, 2), alteration of embryonical growth, or fetal development at multiple stages of gestation (3, 4), and it may even lead to pregnancy loss (5, 6), mainly of these malnutrition deficiencies, which occurs during the periparturient period. There are several common nutritional strategies to improve reproduction in dairy cows without any adverse effect on lactation efficiency, such as feeding high-quality forages, supplementation with amino acids (AA, especially in a rumen-protected form), direct-fed microbial feed during the transition period, or supplemental fats in the diet which are one of the most popular ways to improve energy intake in dairy cows. Amino acid nutrition to dairy cattle is recognized as one of the promising nutritional strategies that have an essential role in reproduction, in addition to the benefit of milk production and other performance aspects. Studies have been proved that several AAs are concentrated in the oviductal and uterine histotroph and the amniotic and allantoic fluids, in comparison with circulating AA concentrations, and many studies have proposed a significant role for these increased AA concentrations in normal embryonic growth and fetal development (7, 8). Methionine (Met) is an essential amino acid (EAA) that could limit reproduction in milking dairy cows (9), in addition to lysine and arginine as well. In mammals, a Met codon is used to initiate most protein synthesis leading to a fundamental role for this AA in all portions of mammalian cellular functions (10, 11).

Studies have evaluated the effects of supplied rumen-protected amino acids (RPAAs), such as rumen-protected lysine (RPL) and rumen-protected methionine (RPM), on milk production and composition; some indicated an increase in milk yield, milk protein percentage, and usually, milk protein yield (12–18), often but not general. For reproduction, lactating cows' diets supplied with RPM or RPL have had positive reproductive outcomes (9, 19) or minor or no effects on reproductive outcomes (20, 21). Some studies have correlated Met concentrations with optimal development in the early embryonic period (22, 23). In addition, the recent studies in both sheep (24, 25) and cattle (7, 26) have confirmed that Met is concentrated in uterine and embryonic fluids, emphasizing the role of increased uterine Met in normal embryonic development and survival. Despite these studies that associate Met and Lys with milk production and reproductive processes, to our knowledge, no previous studies have assessed the effects of feeding a combination of RPL and Met on fertility and pregnancy rate in lactating Holstein dairy cows.

Research on transition and lactating dairy and beef cows (13, 14, 27, 28) has reported improvement in health, milk production, animal performance, and liver function, as well as subsequent offspring due to the dietary provision of RPM and RPL, in some cases but not all. This study hypothesized that supplementing either RPL or RPM or their combination to transition cows and continual feeding their combination during the high milking period would enhance the production and fertility in lactating dairy cows. We specifically hypothesized that RPL and RPM feeding would increase milk protein content and production, as observed in this study during the transition period (18, 29), leading to improved nitrogen utilization. Further, we hypothesized that providing a combination of RPL and RPM would accelerate cows to be conceived, this leads to an increase in the conception rate, and therefore, there would be an increase in the pregnancy rate. The objectives were to evaluate the effects of daily TMR feeding with a combination of RPL and RPM on milk production and composition, nitrogen efficiency, and pregnancy rate.

The results have been presented in partial form during the 2021 Annual Meeting of the American Dairy Science Association (ADSA), Abstract No# 245 “Supplementing ruminally protected methionine or lysine improved milk production in transition cows” and (18, 29, 30).

## Materials and Methods

### Experimental Design and Animals

This research is a part of a large project to study the effect of ruminally protected amino acids on transition dairy cows and their neonatal calves. Cows, calves management, sampling, analysis procedures, and results were previously reported (18, 29, 30). Briefly, this study lasted from 3 weeks before calving until 5 months after calving (approximately 6 months), the study on the dairy cows contained two majors stages; (1) transition period (3 weeks before through 3 weeks after calving), (2) high milking period (from 22 to 150 days in milk), besides the trial on neonatal calves born from the transition cows that were used in this study. During the transition period, a total of 120 late-pregnant Holstein dairy cows were used, and the cows were distributed into eight groups (*n* = 15), and two groups were assigned to each of four experimental treatments (*n* = 30 cows/treatment). Cows were fed either the control diet (CON), or RPL, or RPM or two in combination (RPML). Cows were selected and assigned to treatment based on the days of pregnancy (250 ± 2 days, *p* = 0.84), previous lactation milk production (11,512 ± 1,837 kg; 305-day milk yield, *p* = 0.90), parity (3.09 ± 1.56, *p* = 0.94), and body condition score (BCS; 3.58 ± 0.26, *P* = 0.86). The experiment was conducted as a completely randomized design with treatments arranged in a 2 × 2 factorial. The cows were fed their respective diets starting at about 3 weeks [transition period, (25.0 ± 3.31 days) before the anticipated calving to 3 weeks post-calving (24.0 ± 3.31 days)]. A statistical power analysis was performed before the study using G–power 3.1 software (31), and power = 0.95, α = 0.05, and 0.32 effect size indicated a minimum group size of 100 cows, more details in our previous studies (18, 30). Until the end of the first stage of the study (about 21 DIM), some cows were culled, diseased, sold, or died, and the cows removed from the herd for any reason were excluded from the statistical analysis [refer to our previous paper of Elsaadawy et al. (18)].

In the second stage of the study (during the high milking period), a total of 100 dairy cows have completed the transition period and continued and entered the second stage of the study (25 cows/treatment). The cows were distributed during the high milking period based on their treatment during the transition period as follows: CON**–**RPML, RPM**–**RPML, RPL**–**RPML, and RPML**–**RPML. All cows were fed a combination of RPM and RPL at the rate of 0.17% RPM and 0.41% RPL of DM during the second stage of the study. During the period between 22 and 150 DIM, some cows were culled, diseased, sold, or died, and the cows removed from the herd for any reason were excluded from the statistical analysis (details in the RESULT Section).

Cows were fed total mixed rations (TMRs) *ad libitum* four times per day at 06:00, 12:00, 18:00, and 24:00 h and milked in the automatic rotary parlor four times per day just prior to being fed, at about 6-h intervals. Feed offered was managed to achieve around 5% refusal each day. The diet's TMR composition, chemical analyses, and EAA profile were illustrated (Tables 1–3). Cows were housed in a ventilated, four-row, free-stall barn (center feed alley with two rows of stalls on each side). After calving, they were moved to the colostrum barn for 1–3 days and then relocated to the milking barn. Manure was removed by mechanical scraper 4 times per day when the cows were at the milking parlor at 06:00, 12:00, 18:00, and 24:00 h. Sand bedding was groomed four times/day, and new sand was added two times/day at 06:00 and 12:00 h. Cows had access to *ad libitum* freshwater during the trial. The trial was conducted at the AustAsia Dairy Group, Shandong, China. Temperature humidity index (THI) was measured for the cows' barns using humidity detector PCE-HT 112 (PCE Instruments Ltd, UK). The temperature humidity index was calculated according to the equation of the National Research Council (32).

**Table 1:** Ingredients of the total mixed ration (TMR) fed to Holstein dairy cows during the high milking periods^1^.

**Nutrients (% DM)**	**High milking cow**			
	**CON-RPML**	**RPM-RPML**	**RPL-RPML**	**RPML-RPML**
Corn silage^2^	35.70	35.70	35.70	35.70
Alfalfa hay^3^	8.09	8.09	8.09	8.09
Corn grain flaked^4^	12.70	12.70	12.70	12.70
Corn grain fine	11.33	11.33	11.33	11.33
Soybean meal^5^	6.69	6.69	6.69	6.69
Canola mealsolvent^6^	1.44	1.44	1.44	1.44
Cottonseed fuzzy	4.19	4.19	4.19	4.19
Molasses cane^7^	7.50	7.50	7.50	7.50
Corn gluten meal^8^	2.57	2.57	2.57	2.57
Brewers grains^9^	0.64	0.64	0.64	0.64
High cow premix^10^	5.09	5.09	5.09	5.09
Berga fat 100^11^	1.96	1.96	1.96	1.96
Willmar^12^	1.52	1.52	1.52	1.52
Methionine,^13^	0.17	0.17	0.17	0.17
Lysine^14^	0.41	0.41	0.41	0.41

1 *High milking diets (from 22–150 days in milk), all cow diets of CON-RPML, RPM-RPML, RPL-RPML, and RPML-RPML, were provided with the combination of Met and Lys at a rate of (RPM; 0.17% DM& RPL; 0.41% DM, and NE_L_ = 1.80 Mcal/kg DM)*.

2 *Corn silage contained 32% DM,8.7% CP and 38.52%aNDF*.

3 *Alfalfa hay contained 91.3% DM,21.4% CP and 37.89%aNDF*.

4 *Corn grain flaked contained 86.1% DM and 8.8% CP*.

5 *Solvent soybean meal contained 86.7% DM and 47.5% CP*.

6 *Canola meal Solvent contained 87.5% DM and 42.5% CP*.

7 *Molasses sugarcane contained 60.5% DM and 4% CP*.

8 *Corn gluten meal contained 91.8% DM and 64.6% CP*.

9 *Brewers grains wet contained 22% DM and 31.67% CP*.

10 *High milking cow premix contained (mineral; Na, Cl, Ca, P, Mg, K, and S), (vitamin A, D, K), chelated mineral (Zn, Cu, Se, Co), rumensin, probiotics, and antitoxins*.

11 *Berga Fat100, rumen-protected fat (Berga and Schmidt Nutrition Sdn. Bhd., Malaysia)*.

12 *Willmar (Volac Willmar feed ingredients Ltd., UK): ruminally protected fats*.

13 *Rumen-protected Met (Meta Smart dry, Adisseo, France)*.

14 *Rumen-protected Lys (KeminLysiPEARl, Kemin industries, USA)*.

**Table 2:** Chemical composition of the total mixed ration (TMR) fed to Holstein dairy cows during the High milking periods^1^.

**Nutrients (% DM)**	**High milking cow**			
	**CON-RPML**	**RPM-RPML**	**RPL-RPML**	**RPML-RPML**
NE_L_ (Mcal/kg DM)	1.80	1.80	1.80	1.80
NFC	42.29	42.29	42.29	42.29
Starch	29.32	29.32	29.32	29.32
Ether extract	7.00	7.00	7.00	7.00
NDF	28.01	28.01	28.01	28.01
CP	15.93	15.93	15.93	15.93
ADF	17.91	17.91	17.91	17.91
Ash	6.78	6.78	6.78	6.78
PeNDF	22.27	22.27	22.27	22.27
RDP^*^	9.41	9.48	9.46	9.25
RUP^*^	6.52	6.52	6.49	6.52
RDP supplied (g/day)^*^	2,425	2,399	2,404	2,498
RUP supplied (g/day)^*^	1,681	1,644	1,650	1,788
MP supplied (g/day)^*^	2,942	2,886	2,897	3,095
MP balance (g/day)^*^	218	106	121	131
MP from bacteria (g/day)^*^	1,549	1,526	1,531	1,610
MP from RUP (g/day)^*^	1,392	1,361	1,365	1,486
LYS (g)^*^	203.07	199.7	199.7	214.07
Lys (% MP)^*^	6.90	6.92	6.92	6.92
Met (g/day)^*^	75.18	73.98	73.98	78.38
Met (% MP)^*^	2.56	2.56	2.56	2.53
Lys: Met^*^	2.70	2.70	2.70	2.70
Forage%	43.81	43.81	43.81	43.81
DM	51.71	51.71	51.71	51.71
DCAD (meq/kg DM)	253	253	253	253

1 *High milking diets (from 22 to 150 days in milk), all cow diets of CON-RPML, RPM-RPML, RPL-RPML, and RPML-RPML, were provided with the combination of Met and Lys at a rate of (RPM; 0.17% DM& RPL; 0.41% DM, and NE_L_ = 1.80 Mcal/kg DM)*.

* *Amount of RDP, RUP, and MP was based on the calculation of CNCPS v6.5*.

**Table 3:** Duodenal flows of the indispensable amino acids (IAA) based on the actual consumed DMI during high milking period^1^ using CNCPS v6.5.

**(IAA)**	**High milking diets**							
	**CON-RPML**		**RPM-RPML**		**RPL-RPML**		**RPML-RPML**	
	**AA flow,** **g/day**	**MP,** **%**	**AA flow,** **g/day**	**MP, %**	**AA flow,** **g/day**	**MP, %**	**AA flow,** **g/day**	**MP,** **%**
ARG	221.2	6.29	217.3	6.29	218.0	6.29	232.1	6.28
HIS	91.6	2.54	89.9	2.54	90.2	2.54	96.1	2.54
ILE	178.8	4.89	175.7	4.89	176.3	4.89	187.3	4.87
LEU	313.7	8.47	307.9	8.47	308.9	8.47	329.8	8.48
LYS	249.7	6.90	245.6	6.92	246.5	6.92	262.8	6.92
MET	104.6	2.56	103.1	2.56	103.4	2.56	108.6	2.53
PHE	187.6	5.03	184.2	5.03	184.9	5.03	197.0	5.03
THR	166.7	4.64	163.8	4.64	164.3	4.64	174.7	4.63
TRP	50.8	1.30	49.9	1.30	50.1	1.30	53.2	1.29
VAL	199.9	5.38	196.4	5.38	197.1	5.38	209.6	5.37

1 *High milking diets (from 22 to 150 days in milk), all cow diets of CON-RPML, RPM-RPML, RPL-RPML, and RPML-RPML, were provided with the combination of Met and Lys at a rate of (RPM; 0.17% DM& RPL; 0.41% DM, and NE_L_ = 1.80 Mcal/kg DM)*.


$$\begin{array}{l} \text{THI }=\;\left(1.8\;\times \text{ Tdb }+\;32\right)\;-\;\left(0.55\;-\;0.0055\;\times \text{ RH}\right)\\ \;\times \;\left(1.8\;\times \text{Tdb }-\;26\right) \end{array}$$

where Tdb–dry bulb temperature (°C) and RH—relative humidity (%).

### Ration Formulation

As discussed in our previous studies (18, 30), TMR was formulated using Cornell net carbohydrate and protein system [(CNCPS) v.6.5.5 (33)] as executed in AMTS.Cattle.Professional v.4.7.2 (2016, AMTS LLC, USA) to meet or exceed the requirements of dairy cows (Tables 1, 2). The high-lactating diet was formulated for cows at 50 DIM, 600 kg of BW, 3.25 BCS, producing 55 kg/day milk with a target of 4.0% of milk fat and 3.16% of true protein, and a predicted DMI of 26 kg/day. The dietary content of Met and Lys was balanced according to the recommendations of CNCPS v6.5; to be within 6.15%−7.2% MP-Lys and 2.1%−2.35% MP-Met to meet the Lys-to-Met ratio within the range of 2.7:1 to 3:1. Ruminally protected amino acids were mixed with the premix using a mixer and then top-dressed on the TMR and mixed into the TMR 1 time/day at 06:00 h during the experiment using a Supreme Vertical Feed Mixer (Supreme International Limited, USA). The Goke intelligent feeding system with measurable feed intakes was used (GokeAgri, Beijing, China). Dietary was provided with RPL and RPM at 0.41 and 0.17% of DM, respectively (Tables 1, 2).

The isopropyl ester of 2-hydroxy-4-(methylthio)-butanoic acid (HMBi) was provided in a dry powder form (MetaSmart, Adisseo, France). According to the manufacturer, the product contains 57% HMBi, which is 78% Met equivalency, and 50% of it is absorbed *via* the rumen wall (34). Thus, each gram of the product gives 0.22 g of metabolizable Met. The RPL was provided as a dry powder containing 47.5% l-Lys monohydrochloride (3.2.3) with 70% of bioavailability, according to the manufacturer. Thus, each gram of the product provided 0.33 g of metabolizable Lys-HCl (LysiPEARL, Kemin Industries, USA).

### Sampling, Measurements, and Analysis

#### Feed and Physically Effective NDF

Analysis techniques have been described in detail in our previous study (18). In general, feed offered and refusal were measured daily for each treatment. Diets and the major dietary ingredients were sampled and analyzed weekly and used to calculate nutrient concentrations. Feed samples were dried at 105°C for 4 h to determine the DM percentage and stored at **–**20°C for further analysis. The dried feed samples were ground through a 1-mm screen before analysis using a Cyclotec 1093 Mill (Tecator 1093, Tecator AB, Höganäs, Sweden). Feed samples were further dried at 105°C for 2 h to determine the absolute DM. Chemical analyses of dry matter (DM), crude protein (CP), ether extract (EE), neutral detergent fiber (aNDF), and acid detergent fiber (ADF) were performed using wet chemistry techniques at the State Key Laboratory of Animal Nutrition, Institute of Animal Sciences of Chinese Academy of Agricultural Sciences (CAAS), Beijing, China.

The content of CP (N × 6.25) in feed samples was analyzed using the macro-Kjeldahl nitrogen test [AOAC International, (35); method 984.13.4.09] with a Kjeltec digester 20 and a Kjeltec System 1026 distilling unit (Tecator AB). Ether extract content was analyzed using a soxhlet HT6 apparatus (Tecator AB) according to AOAC International (2000; 920.39), and ADF and aNDF were analyzed according to Van Soest et al. (36) using alpha-amylase with the addition of sodium sulfite. Ash content was analyzed by incineration at 550°C for 5 h, and the organic matter (OM) content was calculated by subtracting ash from 100. Non-fiber carbohydrates (NFC) were calculated by the difference according to the National Research Council (17).

Energy balance (EB), dietary energy density, and MP balance were determined based on the actual DMI of individual ingredients and their respective energy value and MP based on the CNCPS feed library.


$$\begin{array}{l} \text{Net energy intake }\left(\text{NEI}\right)\;=\text{daily DMI }\times \text{ N}{\text{E}}_{\text{L}}\text{ density of the diet}. \end{array}$$

Requirements for net energy (NE) fractions were calculated as


$$\begin{array}{l} \text{Net energy of maintenance }\left(\text{N}{\text{E}}_{\text{M}},\text{ Mcal}/\text{day}\right)\;=\text{B}{\text{W}}^{0.75}\\ \;\times \;0.080. \end{array}$$

The requirement for net energy of lactation (NE_L_, Mcal/day):


$$\begin{array}{l} \text{N}{\text{E}}_{\text{L}}=\;(0.0929\;\times \text{ fat }\%\;+\;0.0547\;\times \text{ protein }\%\;+\;0.0395\\ \;\times \text{ lactose }\%)\;\times \text{ milk production}. \end{array}$$

The net energy requirement for pregnancy (NE_Y_; Mcal/day) was calculated as follows


$$\begin{array}{l} \text{N}{\text{E}}_{\text{Y}}=\;[(0.00318\;\times \text{ day of gestation}-0.0352)\;\\ \;\times (\text{calf birth weight}/45)]/0.218\text{ for animals that were }\\ \;>210-\text{day pregnant},\text{i}.\text{e}.,\text{the pre}-\text{fresh group}. \end{array}$$

The sum of individual requirements was as follows:


$$\begin{array}{l} \text{N}{\text{E}}_{\text{REQ}}\;\left(\text{Mcal}/\text{day}\right)\;=\text{ N}{\text{E}}_{\text{M}}\;+\text{ N}{\text{E}}_{\text{L}}\;+\text{N}{\text{E}}_{\text{Y}} \end{array}$$

Energy balance (EB, Mcal/day) was calculated as the following equation:


$$\begin{array}{l} \text{EB }\left(\text{Mcal}/\text{day}\right)\;=NEI-\;\left(NE_{L}\;+NE_{M}\;+NE_{Y}\right).\\ \text{Supply}/\text{ requirements }=\left[\text{NEI}/\;\left(\text{N}{\text{E}}_{\text{L}}\;+\text{ N}{\text{E}}_{\text{M}}\;+\text{ N}{\text{E}}_{\text{Y}}\right)\right]\\ \;\times \;100. \end{array}$$

Energy corrected milk (ECM 3.5% fat) and fat-corrected milk (FCM) were calculated according to (37), as the following equations:


$$\begin{array}{l} 3.5\text{\% ECM }\left(\text{kg}/\text{day}\right)\;=\left[12.82\;\times \text{ fat yield }\left(\text{kg}\right)\right]\\ \;+\left[7.13\;\times \text{ protein yield }\left(\text{kg}\right)\right]\\ \;+\left[0.323\;\times \text{milk yield }\left(\text{kg}\right)\right]\\ \text{ FCM }\left(\text{kg}/\text{d}\right)\;=\left[0.4324\;\times \text{ of milk yield }\left(\text{kg}\right)\right]\\ \;+\left[16.23\;\times \text{ of milk fat }\left(\text{kg}\right)\right]. \end{array}$$

Physical effective NDF of the TMR (% of DM) was determined weekly using a Penn State Particle Separator (PSPS) sieve, which contains four sieve layers, upper (19-mm pore size), middle (8-mm pore size), lower (4-mm pore size) sieves, and the pan, and the peNDF content of the TMR was estimated by multiplying the NDF content of the feed by the percent of feed retained on PSPS sieve according to the previous description (38).

### Blood Samples

Duplicate blood samples of approximately 15 ml were collected *via* a coccygeal vessel from individual cows on 21, 30, 60, 90, 120, and 150 DIM. Samples were collected into evacuated tubes containing clot activators of silica particles for serum separation (Jiangsu Kangjian Medical Apparatus Co., Ltd, China). Blood samples were then centrifuged at 3,000 × *g* for 15 min at 4°C for separation of the serum, and the supernates were stored at −20°C until further analysis. Blood BHB concentrations were measured immediately after collecting the blood using the BHBCheck Plus blood ketone test (PortaCheck, Inc. Moorestown, NJ, USA). Serum total protein concentrations were analyzed using a digital temperature-compensating refractometer (Model 300027, SPER Scientific Ltd., Scottsdale, Arizona, USA). Before testing each sample, the refractometer prism was cleaned and the refractometer was calibrated with distilled water.

### Milk Samples and Income Over Cost Calculation

Milk sampling has been detailed in our previous paper (18). Briefly, milk samples were collected on 21, 30, 60, 90, 120, and 150 DIM. Each milk sample was preserved with bronopol-B2 preservative (D&F Control Systems Inc., Dublin, ON, Canada) and stored at 4°C with subsequent analysis for milk fat, total protein, lactose, total solids (TS), and milk urea nitrogen (MUN) using a mid-infrared machine (MilkoScan FT3; Foss-600, Foss Analytics, Hillerød, Denmark). Somatic cell count (SCC) was analyzed using a Somatic Cells Analyzer (Foss, Hillerød, Denmark). Somatic cell score (SCS) was then obtained by transforming SCC using the equation (39). Daily milk production for individual cows was automatically recorded using parlor milk weight sensors, and data were transferred to the dairy herd management software [(DelPro™ Software v5.4 (DeLaval Corporation, Sweden)]. Daily data were then summarized as average weekly and monthly data points for statistical analysis.

Milk price was calculated using the milk price of $19.78/cwt (cwt = hundredweight of milk), protein price of $7.12/lb, and fat price of $1.97/lb [U.S. Department Of Agriculture (USDA): Announcement of Advanced Pricing and Pricing Factors for August-2020] https://www.ams.usda.gov/mnreports/dymadvancedprices.pdf and protein yield and fat yield corresponding to each treatment.


$$\begin{array}{l} \text{Milk income}=\left(\text{average milk yield }\times \text{ milk price}/100\right)\\ +\;\left(\text{milk protein yield }\times \text{ protein price}\right)\\ +\;\left(\text{milk fat yield }\times \text{ fat price}\right)\text{for each treatment group}.\\ \text{Income over feed cost }\left(\text{IOFC}\right)=\left(\text{milk income}-\text{feed cost}\right). \end{array}$$

The cost of the feed per each 1 kg DMI was calculated according to the actual purchase prices of each feedstuff ingredient during the experiment.

### Body Condition Score

The evaluation of BCS has been described in detail in our previous study (18). Briefly, BCS was assessed for individual cows by the same two trained professionals on 21, 30, 60, 90, 120, and 150 DIM. A combination of visual appraisal and manual palpation of different body areas (between the hooks, hooks and pins, tailhead to pins, transverse processes, and rumen fill) was evaluated on a 1–5-point scale (17).

### Fertility Performance

Reproductive parameters of DIM at 1st insemination, heat detection rate (HDR), conception rate (CR), and pregnancy rate (PR) were obtained by DelPro™ 5.4 Herd Management Software (DeLaval Corporation, Sweden). Cows on all treatments had a voluntary waiting period of 56 days post-calving. A double Ovsynch protocol was performed, which proceeded through two OvSynch protocols 7 days apart and was followed by timed artificial insemination (TAI) after the second protocol (40). Ultrasonography checking of the ovaries was conducted as a regular procedure in the reproduction management of dairy cattle herds using a portable ultrasound fitted with a 7.5 MHz linear array transducer (Shenzhen Bondway Electronics Co., Ltd, Shenzhen, China). Pregnancy diagnosis was performed using ultrasonography to evaluate embryonic and amniotic vesicle size to detect a viable conceptus on day 32 after artificial insemination (AI). Cows diagnosed as pregnant on day 32 were rechecked on days 47 and 65. The DIM at 1st insemination was defined as the days between calving and the first service. Heat detection rate is the percentage of cows inseminated over a 21-day period divided by the number of cows eligible to be bred over those 21 days. Conception rate is defined as the percentage of pregnant cows divided by the number of cows inseminated during 21 days. Pregnancy rate (21-day interval) was calculated by multiplying the heat detection rate by the conception rate [DelPro™ 5.4 Herd Management Software (DeLaval Corporation, Sweden)].

### Statistical Analyses

The high milking period was restricted from 22 to 150 DIM. Daily DMI and milk yield were condensed to weekly and monthly averages. Data were analyzed using a model with treatment effects (RPL and RPM), time, and their interaction using PROC MIXED of SAS (v. 9.4, SAS Institute Inc., USA, 2013). The MIXED statistical model used for analysis was as follows:


$$\begin{array}{l} {\text{Y}}_{\text{ijk}}=\text{μ}+{\text{D}}_{\text{i}}\;+{\text{A}}_{\text{ij}}+{\text{T}}_{\text{k}}\;+\text{D}{\text{T}}_{\text{ik}}+{\text{ε}}_{\text{ijk}}, \end{array}$$

where Y_ijk_ was the dependent, continuous variable, μ was the overall mean; D_i_ was the fixed effect of diet (i = 1, 2, 3, or 4); A_ij_ was the random effect of the jth cow within the ith treatment; T_k_ was the fixed effect of time (day); DT_ik_ was the interaction effect of diet and time; and ε_ijk_ was the residual error. The Kenward-Roger option was used for computing the denominator degrees of freedom for testing hypotheses (41). The distribution of the residuals was assessed to determine normality and homoscedasticity. A log transformation was used for the milk SCC variable to convert it to SCS and to enhance the homogeneity of the distribution of residuals.

Fertility data were analyzed by chi-square test using SAS PROC FREQ procedures. SAS linear regression with PROC REG procedure was conducted to indicate the relationship between either peak of milk production or DIM at the peak of milk (dependent variable), and average DMI consumed during the fresh period (between 1 and 21 DIM, independent variable), with 95% prediction intervals that were calculated for various DMI, DIM, and milk yield.

Pearson correlation analysis using SAS PROC CORR procedure was performed to study the relationship between the consumed DMI during the fresh period (first 3 weeks of lactation) and the peak of milk production and DIM at the peak of milk for dairy cows fed either RPL or RPM or the combination throughout the transition period. The least-squares means were compared using the least significant difference (LSD), and statistical differences were declared significant at *p* ≤ 0.05. A tendency was determined at *p* > 0.05 to *p* ≤ 0.10.

## Results

### Dry Matter Intake, Barn Characteristics, Cow Exclusions, and Pen State Particle Separator

**Figure 1:**
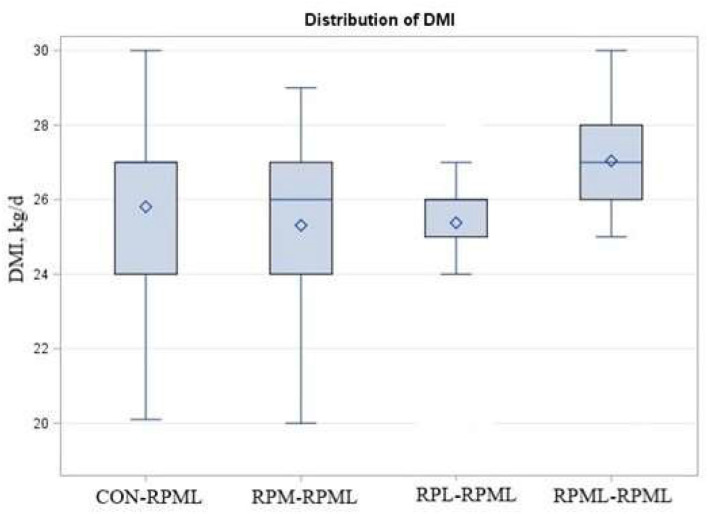
Effect of supply ruminally protected Met and Lys to transition and milking dairy cows on dry matter intake. Values are means; standard errors represented by vertical bars.

There were significant Trt × Time interactions (*p* < 0.001), as the DMI of cows that fed RPML**–**RPML supplements was always greater (27 kg DMI, *p* < 0.001) than other treatments at all time points: 25.8, 25.3, and 25.4, for CON**–**RPML, RPM**–**RPML, and RPL**–**RPML, respectively (Table 4, Figure 1). There were time effects for DMI, in which DMI was higher at 90 and 120 DIM (26.7 kg) than other time points 24.03, 25.8, and 26.1 kg for feed consumption at 30, 60, and 150 DIM, respectively (*p* < 0.001). The physical characteristics of high milking TMR were (DM bases, mean ± SD) 10.9 ± 2.5% on upper, 36.9 ± 2.1% on middle, 19.4 ± 1.3% on lower sieves, and 32.8 ± 2.8% in the pan. The average THI was 56.3 ± 4.2 for the barn during the high milking period. A total of five cows were excluded from the statistical analysis during the second stage of the study due to lameness (RPML**–**RPML, *n* = 1, at 84 DIM), due to mastitis (CON**–**RPML, *n* = 1, at 144 DIM), breaking (CON**–**RPML, *n* = 1, at 110 DIM), or digestive problems (RPM**–**RPML, *n* = 1, at 138 DIM; and RPL**–**RPML, *n* = 1, at 96 DIM). So, in total, 95 animals were used for the statistical analysis as follows: CON**–**RPML = 23 cows, RPM**–**RPML = 24 cows, RPL**–**RPML = 24 cows, and RPML**–**RPML = 24 cows.

**Table 4:** Feeding ruminally protected Met and Lys to transition dairy cows and its subsequent effect on post-calving performance.

**Variable**	**Treatment** ^**1**^				**SEM^**2**^**	***p*** **-value**		
	**CON L–RPML**	**RPM–RPML**	**RPL–RPML**	**RPMLRPML**		**Trt^**3**^**	**Time^**4**^**	**Trt × time^**5**^**
During the high milking period (22–150 DIM)								
DMI (kg/day)	25.8^b^	25.3^c^	25.4^c^	27.0^a^	0.10	<0.001	<0.001	<0.001
Energy balance (EB, Mcal/day)	3.22	2.39	2.99	3.82	0.50	0.26	<0.001	0.02
BCS	3.32^c^	3.46^b^	3.51^b^	3.63^a^	0.02	<0.001	<0.001	<0.001
BCS Change (from 22 to 150 DIM)	0.68^a^	0.43^b^	0.18^c^	0.20^c^	0.05	<0.001	–	–
BHB (mmol/L)	0.90^a^	0.84^b^	0.77^c^	0.72^c^	0.03	0.002	<0.001	0.004
Serum total protein (Brix)	7.43	7.35	7.11	7.34	0.11	0.19	0.004	0.58
Days to peak of milk (day)	63.1^a^	57.1^ab^	55.6^c^	47.2^d^	2.35	<0.004	–	–
Peak of milk (Kg)	46.4^d^	50.5^c^	52.9^b^	56.2^a^	1.02	<0.001	–	–

a,b,c,d *Mean values with different superscripts in the same row were significantly different (p < 0.05)*.

1 *High milking diets (from 22–150 days in milk), all cow diets of CON-RPML, RPM-RPML, RPL-RPML, and RPML-RPML, were provided with the combination of Met and Lys at a rate of (RPM; 0.17% DM& RPL; 0.41% DM, and NE_L_ = 1.80 Mcal/kg DM)*.

2 *Standard error means of all treatments*.

3 *Trt = effect of treatment*.

4 *Time= effect of time: 21, 30, 60, 90, 120, and 150 relatives to calving day*.

5 *Interaction of treatment × time*.

### Metabolizable Protein Balance and CNCPS Evaluation of Diets

The mean chemical compositions of feed ingredients throughout the experiment were used to evaluate the high milking diets by the CNCPS model. Unlike early lactation, high milking metabolizable protein (MP) balance was positive across all rations. The CON**–**RPML diet provided an average of 112, 97, and 87 g/day more MP balance than either RPM**–**RPML, RPL**–**RPML, and RPML**–**RPML diet, respectively (Table 2).

### Effects of Amino Acids Supply on Energy Balance and Body Condition Score

The main effects of supplementing a combination of Lys and Met during the high milking period on EB, BCS, and BCS changes are presented (Table 4). Energy balance was not differed between treatments during the high milking period (*p* = 0.26). There was a time effect on EB (*p* < 0.001), in which EB at 30 DIM was lower than other time points **–**0.41 vs. 2.5, 3.79, 4.58, and 5.06 Mcal/day for EB at 30, 60, 90, 120, and 150 DIM, respectively (*p* < 0.001), and lower (*p* < 0.05) EB at 60 DIM (2.5 Mcal) in comparison with EB at 120 DIM (4.58 Mcal) and 150 DIM (5.06 Mcal). There were Trt × Time interactions for EB (*p* = 0.02; Table 4), because cows consumed RPL**–**RPML (1.84 Mcal) and RPML**–**RPML (1.44 Mcal) had a greater (*p* < 0.05) EB than those cows that received CON**–**RPML (−2.32 Mcal) or RPM**–**RPML (−2.59 Mcal) at 30 DIM; and a tendency (*p* = 0.09) at 60 DIM for greater EB for RPML**–**RPML (3.69 Mcal) compared with RPM**–**RPML (0.93 Mcal); and cows that fed CON**–**RPML (6.11 Mcal) had a greater (*p* = 0.02) EB than cows in RPL**–**RPML group (2.16 Mcal) at 120 DIM, and also cows in RPML**–**RPML (5.15 Mcal) had a greater EB (*p* = 0.04) than cows in RPL**–**RPML (2.16 Mcal) at 120 DIM; and a tendency (*p* = 0.07) for greater EB for CON**–**RPML (6.94 Mcal) compared with cows in RPM**–**RPML group (3.99 Mcal) at 150 DIM (Table 4).

**Figure 2:**
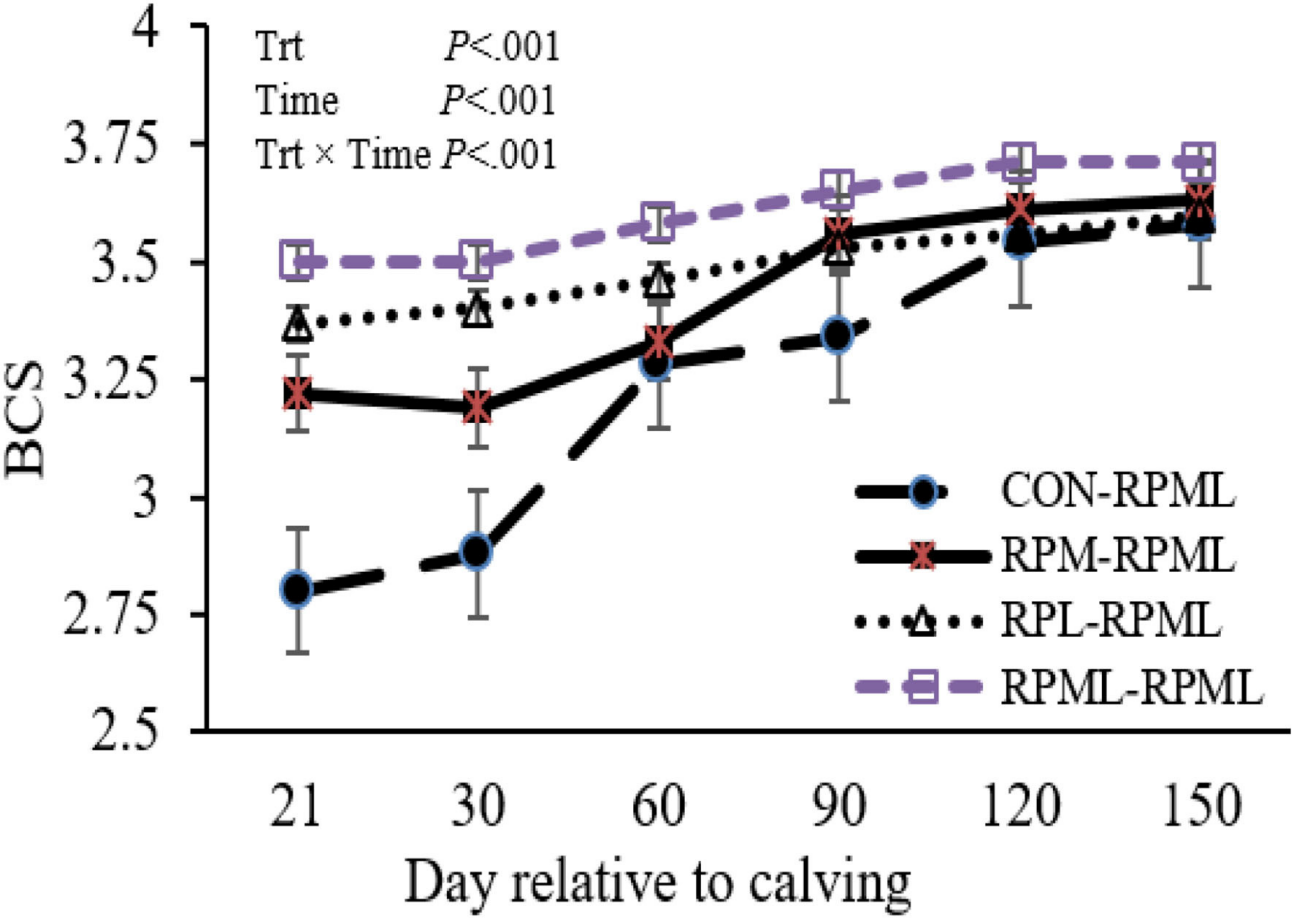
Effect of supply ruminally protected Met and Lys to transition and milking dairy cows on body condition score. Values are means; standard errors are represented by vertical bars.

Transition cows fed supplemental AA diets still had greater BCS than other cows (*p* < 0.001) 3.46, 3.51, 3.63, and 3.32, for RPM**–**RPML, RPL**–**RPML, RPML**–**RPML, and CON**–**RPML, respectively (Table 4, Figure 2). There were time effects for BCS (*p* < 0.001), as the BCS increased with the progression of lactation 3.25, 3.41, 3.52, 3.61, and 3.63 for 30, 60, 90, 120, and 150 DIM, respectively (*p* < 0.001). There were Trt × Time interactions for BCS (*p* < 0.001; Table 4, Figure 2), because cows consumed RPM**–**RPML (3.18) or RPL**–**RPML (3.41) or RPML**–**RPML (3.51) had a greater (*p* < 0.001) BCS than those cows that received CON**–**RPML (2.89) at 30 DIM; and at 60 DIM, cows fed RPL**–**RPML (3.46) or RPML**–**RPML (3.57) diets had a greater (*p* < 0.001) BCS than those cows that fed CON**–**RPML (3.27) at 30 DIM; and at 90 DIM, cows consumed RPM**–**RPML (3.56) or RPL**–**RPML (3.52) or RPML**–**RPML (3.65) had a greater (*p* < 0.001) BCS than those cows that received CON**–**RPML (3.33). The change of BCS from 22 to 150 DIM was affected (*p* < 0.001) by RPAA supply during transition period and that effect was continuing to appear on subsequent lactation during the high milking period and later on.

### Effect of Amino Acids Supply on Days to Peak and Peak of Milk

**Figure 3:**
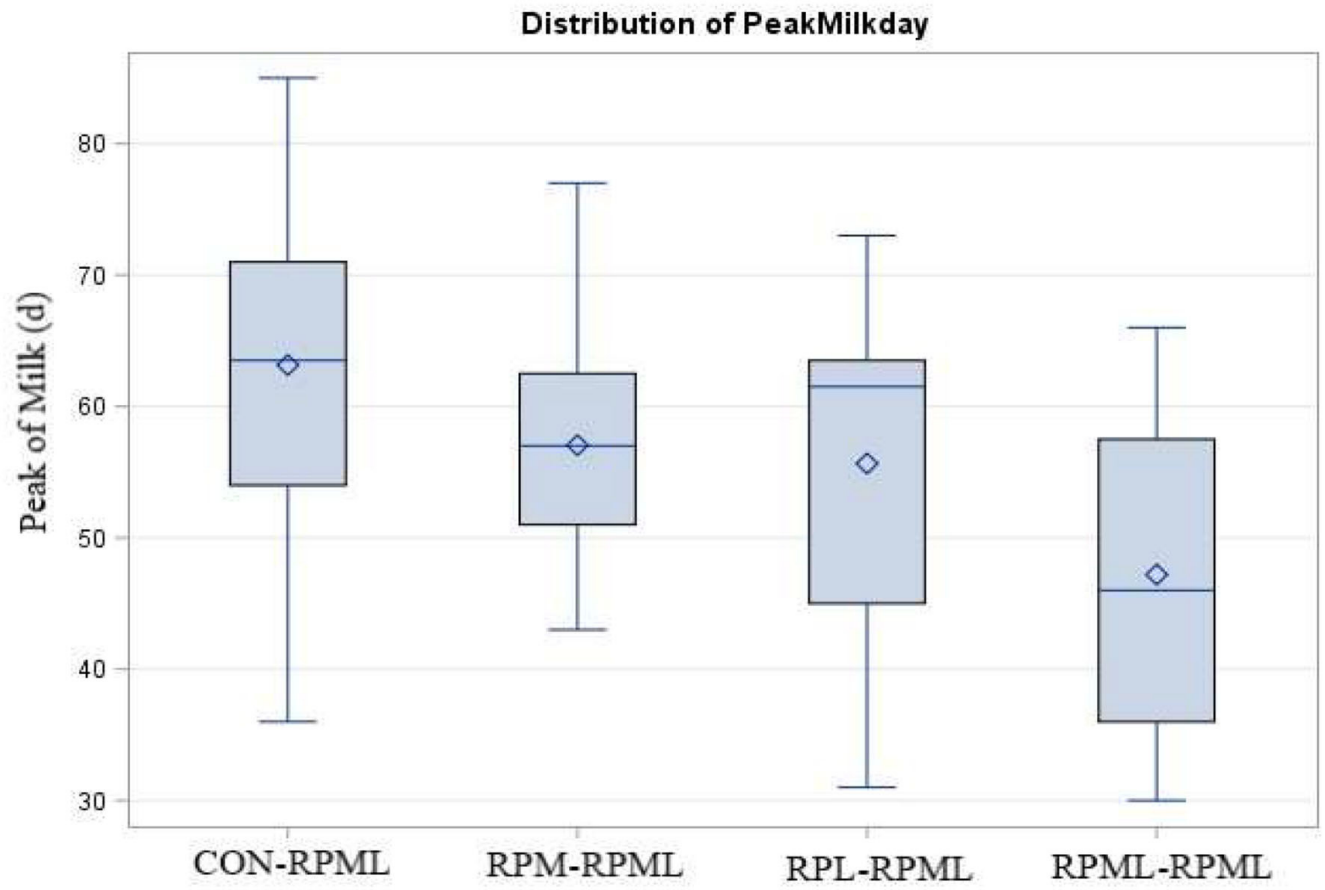
Effect of supply ruminally protected Met and Lys to transition and milking dairy cows on the peak of milk production in days. Values are means; standard errors are represented by vertical bars.

There was a significant (*p* < 0.004) effect of AA supply on days to reach the peak of milk (Table 4, Figure 3), as transition cows fed RPML**–**RPML (47.2 days) quickly (*p* < 0.05) reached the peak of milk than CON**–**RPML (63.1 days), RPM**–**RPML (57.1 days), and RPL**–**RPML (55.6 days); there was a difference (*p* = 0.004) between RPL**–**RPML (55.6 days) and CON**–**RPML (63.1 days); and there was a tendency (*p* = 0.09) between RPM**–**RPML (57.1 days) and CON**–**RPML (63.1 days).

**Figure 4:**
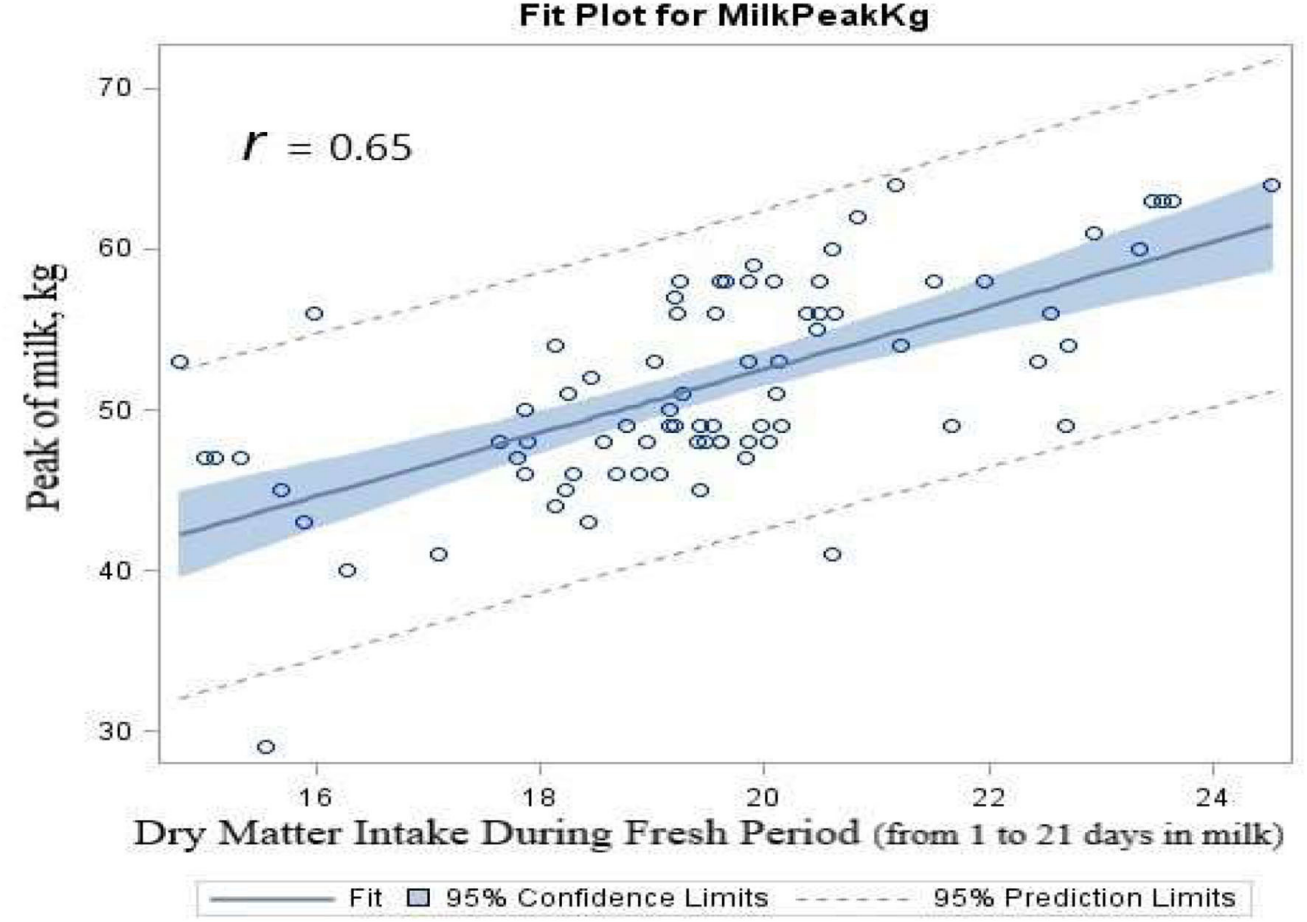
Scatterplot of the peak of milk (kg) by dry matter intake that was consumed during the fresh period (first 3 weeks of lactation) overlaid with the fit line, a 95% confidence band and lower and upper 95% prediction limited, for dairy cows fed either ruminally protected Lys or Met or the combination during the transition period.

The results of regression analysis showed that there were significantly (*p* < 0.0001) positive linear associations between DMI consumed during the fresh period (first 21 DIM) and the peak of milk production (*r* = 0.65, Figure 4). Linear regression of DIM to reach the peak of milk by the average DMI consumed during the fresh period indicated a significance (*r* = 0.41, *p* < 0.0001). The correlation analysis results showed a strong correlation between the average DMI consumed during the first 3 weeks of lactation and the peak of milk production (*r* = 0.67, *p* < 0.0001). There was a negative linear correlation between DMI and DIM to reach the peak of milk (*r* = −0.48, *p* < 0.0001). There was a negative linear correlation between the peak of milk yield and DIM to reach the peak of milk (*r* = −0.63, *p* < 0.0001).

**Figure 5:**
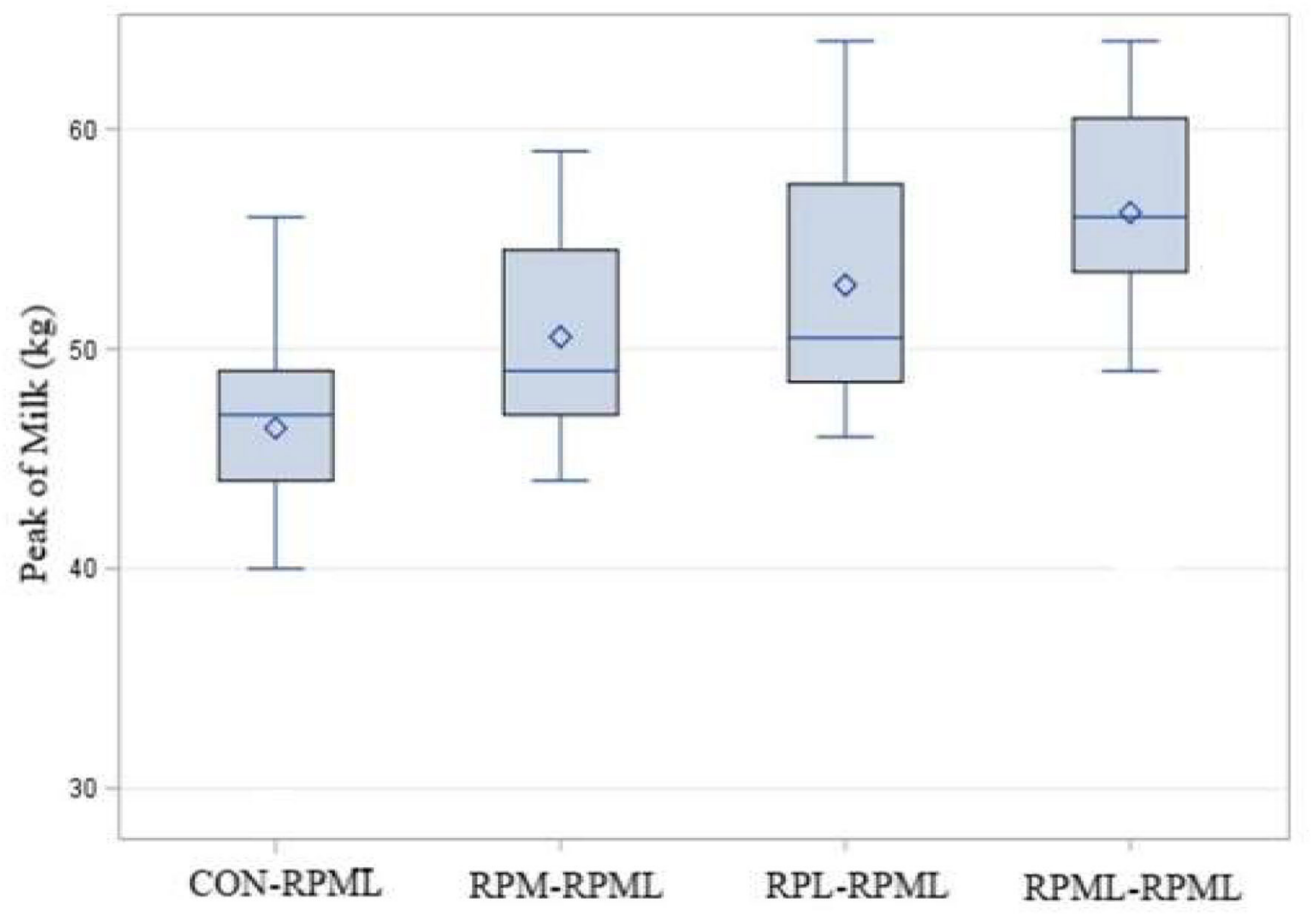
Effect of supply ruminally protected Met and Lys to transition and milking dairy cows on the peak of milk. Values are means; standard errors are represented by vertical bars.

There was a significant (*p* < 0.001) effect of supplementing RPAA on the peak of milk (Table 4, Figure 5), in which transition cows provided RPML**–**RPML diet (56.20 kg/day) produced more (*p* < 0.001) milk yield during the peak of milk in comparisons with CON**–**RPML (46.4 kg/day), RPM**–**RPML (50.5 kg/day), and RPL**–**RPML (52.9 kg/day); there was no difference (*p* > 0.10) between RPM**–**RPML and RPL**–**RPML.

### Effect of Amino Acids Supply on Concentrations of β-Hydroxybutyrate and Serum Total Protein

There was an effect (*p* < 0.002) of RPAA supply on BHB concentration, as cows consumed RPML**–**RPML diet (0.72 mmol/L) had a lower (*p* < 0.05) BHB concentration than cows that fed either CON**–**RPML (0.90 mmol/L) or RPM**–**RPML (0.84 mmol/L), but no difference (*p* > 0.10) between RPL**–**RPML and RPML**–**RPML (Table 4). There was a time effect (*p* < 0.001) for BHB, as the concentration of BHB decreased (*p* < 0.001) with the progression of lactation. There were Trt × Time interactions for BHB concentration (*p* = 0.004; Table 4), as cows fed CON**–**RPML (1.20 mmol/L) had higher (*p* < 0.05) BHB than RPM**–**RPML (0.99 mmol/L), RPL**–**RPML (0.85 mmol/L), and RPML**–**RPML (0.76 mmol/L) at 30 DIM; and a difference (*p* = 0.002) between RPM**–**RPML (0.99 mmol/L) and RPML**–**RPML (0.76 mmol/L) at 30 DIM. Serum total protein concentration did not differ (*p* = 0.19) between the treatments; there were no interactions between Trt × Time for serum total protein concentration (*p* = 0.58). There was a time effect for serum total protein concentration (*p* = 0.004; Table 4), with serum total protein concentration higher (*p* = 0.003) at 150 DIM compared to the other time points.

### Effects of Amino Acids Supply on Milk Production and Composition

**Figure 6:**
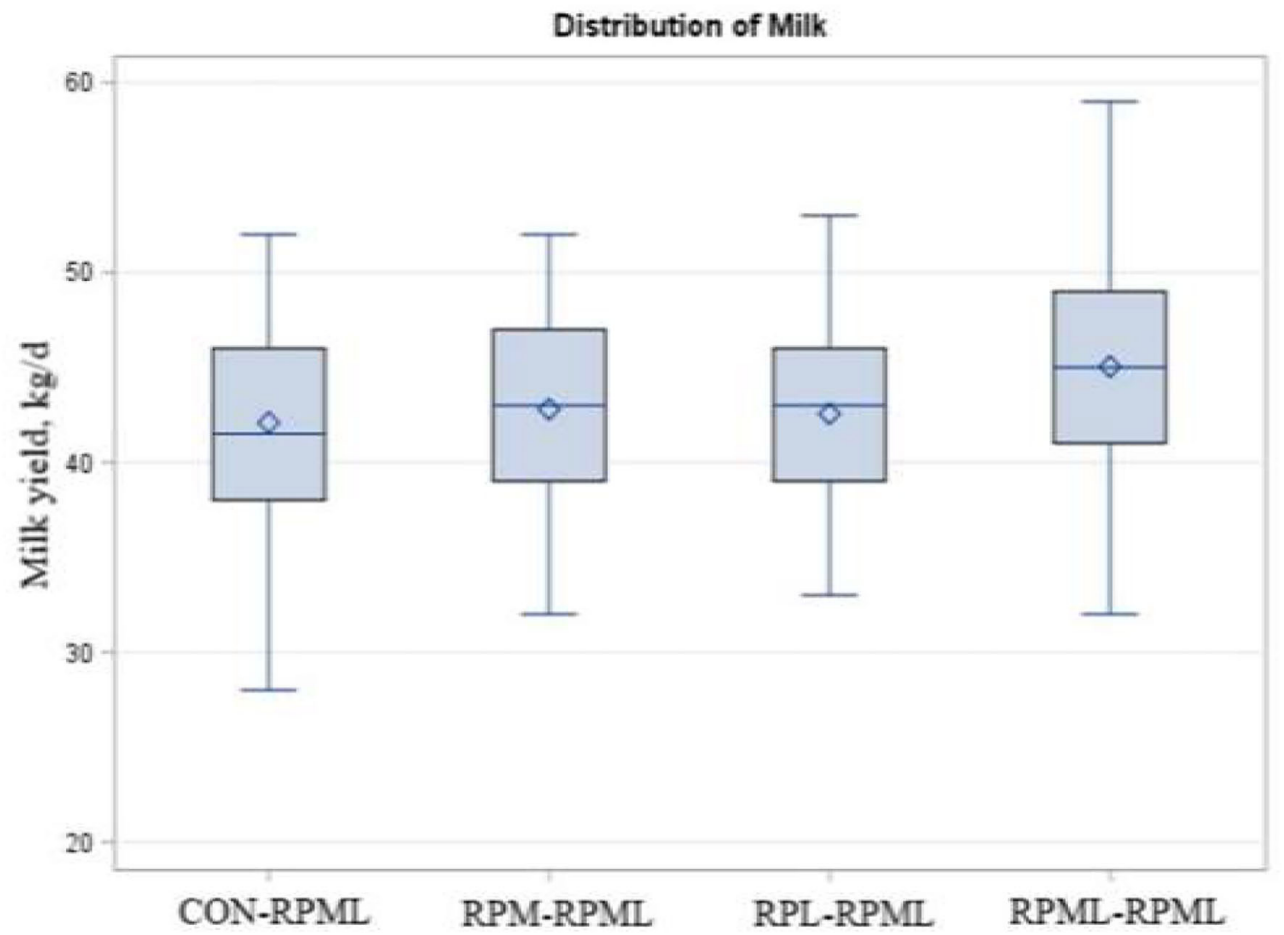
Effect of supply ruminally protected Met and Lys to transition and milking dairy cows on milk yield. Values are means; standard errors are represented by vertical bars.

As indicated in Table 5, Figure 6, transition cows fed RPAA supply are still producing more milk during the high milking period in comparison with transition cows that there were fed the control diet, with cows fed RPML**–**RPML diet (45 kg/day) producing more (*p* = 0.009) milk yield than CON**–**RPML (42.1 kg/day), RPM**–**RPML (42.8 kg/day), and RPL**–**RPML (42.6 kg/day). Time affected milk yield (*p* = 0.001), in which milk yield at 150 DIM, was the lowest (*p* < 0.05) compared with other time points at 30, 60, 90, and 120 DIM.

**Table 5:** Feeding ruminally protected Met and Lys to transition dairy cows and its subsequent effect on post-calving performance.

**Variable**	**Treatment** ^**1**^				**SEM^**2**^**	***p*** **-value**		
	**CON–RPML**	**RPM-RPML**	**RPL–RPML**	**RPML–RPML**		**Trt^**3**^**	**Time^**4**^**	**Trt × time^**5**^**
**Milk production, kg/day**								
Milk yield	42.1^b^	42.8^b^	42.6^b^	45.0^a^	0.62	0.009	0.001	0.66
ECM	44.6^b^	44.5^b^	43.9^b^	46.7^a^	0.77	0.04	0.03	0.36
FCM	45.5^ab^	45.0^ab^	44.2^b^	46.9^a^	0.87	0.08	0.05	0.47
**Milk composition, %**								
Fat	4.03^a^	3.81^b^	3.74^b^	3.77^b^	0.11	0.04	0.29	0.36
Protein	3.15^b^	3.22^ab^	3.22^ab^	3.27^a^	0.05	0.08	0.25	0.13
Lactose	4.84	4.85	4.84	4.86	0.03	0.96	0.92	0.99
Total solid	12.74	12.82	12.67	12.62	0.16	0.86	0.87	0.48
**Milk composition yield, kg/day**								
Fat	1.68	1.63	1.59	1.69	0.05	0.41	0.29	0.41
Protein	1.32^b^	1.37^b^	1.36^b^	1.47^a^	0.02	0.001	0.01	0.03
Lactose	2.03^b^	2.07^b^	2.06^b^	2.19^a^	0.03	0.007	0.001	0.62
Total solid	5.36	5.48	5.39	5.68	0.10	0.12	0.003	0.65
MUN, mg/dl	12.88^a^	11.54^b^	12.09^b^	10.76^c^	0.29	0.002	<0.001	<0.001
Milk SCS^6^	3.55^b^	3.54^b^	3.71^a^	3.62^ab^	0.07	0.09	0.11	<0.001
Feed efficiency, ECM:DMI	1.74	1.77	1.73	1.73	0.03	0.66	<0.001	0.33
Nitrogen Efficiency	31.2^b^	33.3^a^	32.8^a^	33.1^a^	0.62	0.04	<0.001	0.34
IOFC, $/day	10.86^b^	11.42^ab^	11.28^ab^	11.86^a^	2.23	0.10	<0.001	0.95

a,b,c *Mean values with different superscripts in the same row were significantly different (p < 0.05)*.

1 *High milking diets (from 22–150 days in milk), all cow diets of CON-RPML, RPM-RPML, RPL-RPML, and RPML-RPML, were provided with the combination of Met and Lys at a rate of (RPM; 0.17% DM& RPL; 0.41% DM, and NE_L_ = 1.80 Mcal/kg DM)*.

2 *Standard error means of all treatments*.

3 *Trt = effect of treatment*.

4 *Time= effect of time: 21, 30, 60, 90, 120, and 150 relatives to calving day*.

5 *Interaction of treatment × time*.

6 *Milk somatic cell score (SCS)*.

**Figure 7:**
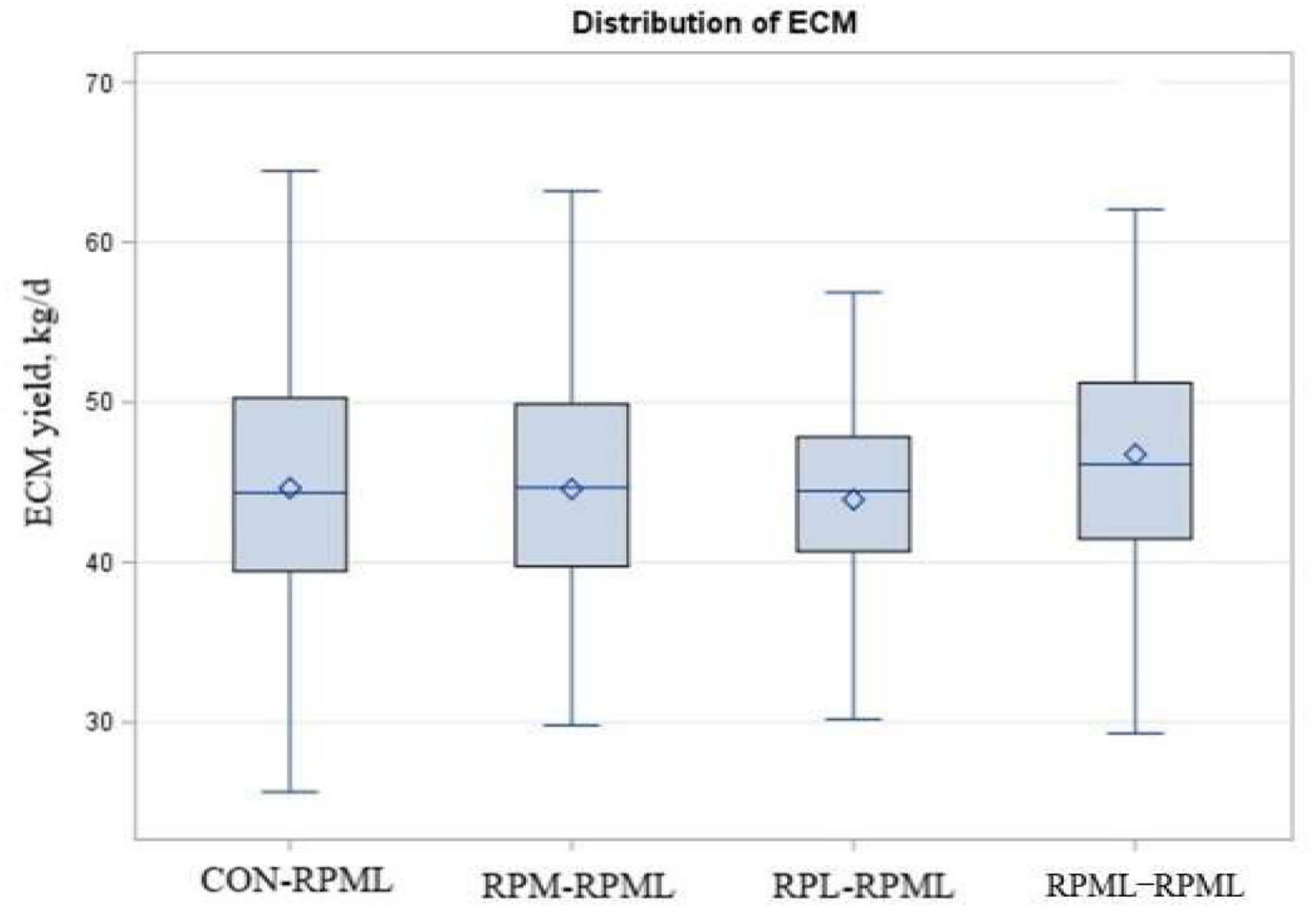
Effect of supply ruminally protected Met and Lys to transition and milking dairy cows on ECM yield during the subsequent lactation. Values are means; standard errors are represented by vertical bars.

As shown in Table 5, Figure 7, energy-corrected milk yield significantly increased (*p* = 0.04) for those cows that received RPML**–**RPML (46.7 kg/day) than CON**–**RPML (44.6 kg/day), RPM**–**RPML (44.5 kg/day), and RPL**–**RPML (43.9 kg/day). Time had an effect on ECM yield (*p* = 0.03), with ECM yield showed the lowest at 150 DIM compared with other time points at 30, 60, 90, and 120 DIM (*p* = 0.01). There were no Trt × Time interactions (*p* = 0.36) for ECM yield (Table 5). Fat-corrected milk tended (*p* = 0.08) to be affected by supplemental AA, with cows fed RPML**–**RPML diet (46.9 kg) produced more (*p* = 0.003) FCM yield than RPL**–**RPML (44.2 kg/day), and cows fed RPML**–**RPML diet (46.9 kg) tended (*p* = 0.10) to increase FCM than CON**–**RPML (45.5 kg) and RPM**–**RPML (45.0 kg). Time affected (*p* = 0.05) FCM yield, with FCM yield, showed the lowest (*p* = 0.01) at 150 DIM compared with other time points at 30, 60, 90, and 120 DIM.

As indicated in Table 5, milk fat content significantly (*p* = 0.04) differs between treatments, in which cows fed CON**–**RPML (4.03 %) had greater milk fat content than RPM**–**RPML (3.81 %), RPL**–**RPML (3.74 %), and RPML**–**RPML (3.77%). As shown also in Table 5, milk protein content responded (*p* = 0.08) to AA supply, as milk produced from cows fed RPML**–**RPML had the higher (*p* = 0.05) milk protein content (3.27%) than CON**–**RPML (3.15%), and tended (*p* = 0.09) to increase than RPM**–**RPML (3.22%) and RPL**–**RPML (3.22%).

As shown in Table 5, milk protein yields positively responded (*p* = 0.001) to the supply of AA, with cows that consumed RPML**–**RPML had a greater (*p* = 0.001) protein yield (1.47 kg) in comparison with CON**–**RPML (1.32 kg), RPM**–**RPML (1.37 kg), and RPL**–**RPML (1.36 kg). There was no difference (*p* > 0.10) in milk protein yield between CON**–**RPML, RPM**–**RPML, and RPM**–**RPML (Table 5). Time had an effect (*p* = 0.01) on milk protein yield, in which protein yield at 150 DIM was the lowest (*p* < 0.05) compared with other time points at 30, 60, and 90 DIM, and a strong tendency (*p* = 0.06) for decrease at 120 DIM. There were Trt × Time interactions for milk protein yield (*p* = 0.03; Table 5), with cows consumed RPML**–**RPML (1.57 kg) which had higher (*p* < 0.05) milk protein yield than CON**–**RPML (1.19 kg), RPM**–**RPML (1.36 kg), and RPL**–**RPML (1.41 kg) at 30 DIM and a higher (*p* = 0.008) milk protein yield in RPML**–**RPML (1.49 kg) than CON**–**RPML (1.29 kg) at 120 DIM; milk protein yield tended (*p* = 0.08) to increase in RPML**–**RPML (1.49 kg) compared to RPM**–**RPML (1.36 kg) at 120 DIM; and there was a tendency (*p* = 0.09) to increase milk protein yield for cows that fed RPL**–**RPML (1.37 kg) in comparison with CON**–**RPML (1.25 kg) at150 DIM.

Lactose yield was affected (*p* = 0.007) by RPAA supply, as cows provided with RPML**–**RPML (2.19 kg) supplementation had a greater (*p* = 0.007) lactose yield compared to CON**–**RPML (2.03 kg), RPM**–**RPML (2.07 kg), and RPL**–**RPML (2.06 kg; Table 5). The difference in lactose yield between CON**–**RPML, RPM**–**RPML, and RPM**–**RPML was insignificant (*p* > 0.10). Time affected lactose yield (*p* = 0.001), as the lowest (*p* < 0.05) lactose yield, produced at 150 DIM compared with other time points at 30, 60, and 90 DIM.

As shown in Table 5, MUN decreased (*p* = 0.002) with RPAA supply, as those cows that were fed diet supplied with RPML**–**RPML (10.76 mg/dl) had a lower (*p* < 0.05) MUN than cows in CON**–**RPML (12.88 mg/dl), RPM**–**RPML (11.54 mg/dl), and RPL**–**RPML (12.09 mg/dl). Milk urea nitrogen was affected by time (*p* < 0.001), as MUN increased linearly (*p* < 0.05) with the progression of lactation. There were Trt × Time interactions for MUN (*p* < 0.001; Table 5), as RPM**–**RPML (11.47 mg/dl) had higher (*p* < 0.05) MUN level than CON**–**RPML (9.54 mg/dl) RPL**–**RPML (9.43 mg/dl) and RPML**–**RPML (9.34 mg/dl) at 60 DIM; and milk from cows that consumed CON**–**RPML diet (15.84 mg/dl) contained a higher (*p* < 0.05) MUN than RPM**–**RPML (9.66 mg/dl), RPL**–**RPML (13.40 mg/dl), and RPML**–**RPML (9.43 mg/dl) at 90 DIM.

Somatic cell count tended (*p* = 0.09) to decrease with supplemental AA (Table 5), with cows that received RPL**–**RPML diet (3.71 log-transformed) had a higher (*p* < 0.05) SCC compared with CON**–**RPML (3.55 log-transformed) and RPM**–**RPML (3.54 log-transformed), and tended (*p* = 0.09) to increase compared with RPML**–**RPML (3.62 log-transformed). There were Trt × Time interactions for SCC (*p* < 0.001), in which cows that consumed RPML**–**RPML (4.05 log-transformed) had higher (*p* < 0.05) SCC at 150 DIM than cows that were fed RPM**–**RPML (3.7 log-transformed) and RPL**–**RPML (3.3 log-transformed).

### Effect of Amino Acids Supply on Feed and Nitrogen Efficiency and Income Over Feed Cost

**Figure 8:**
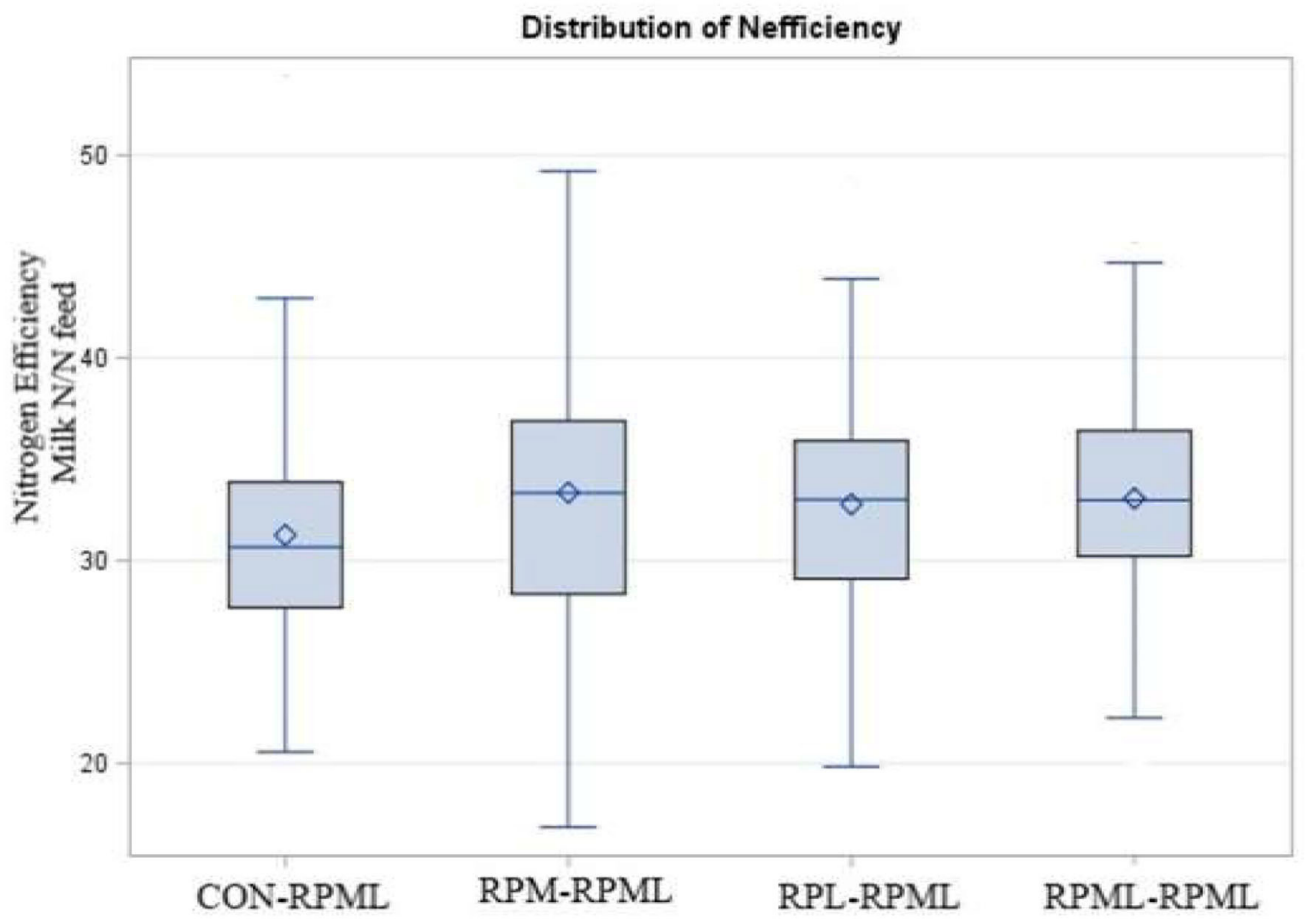
Effect of supply ruminally protected Met and Lys to transition and milking dairy cows on nitrogen efficiency (Milk protein N/N feed intake). Values are means; standard errors are represented by vertical bars.

The effect of AA supply on feed and nitrogen efficiency is shown in Table 5. Feed efficiency was not affected (*p* = 0.66) by AA supplementation. There was a time effect for feed efficiency (*p* < 0.001), in which feed efficiency was higher (*p* < 0.001) at 30 DIM (1.91 ECM: DMI) than feed efficiency at 60 DIM (1.77 ECM: DMI), 90 DIM (1.72 ECM: DMI), 120 DIM (1.69 ECM: DMI), and 150 DIM (1.64 ECM: DMI). Nitrogen efficiency (N efficiency = milk protein N/N feed intake) significantly differed (*p* = 0.04, Figure 8), with a lower (*p* = 0.04) N efficiency for CON**–**RPML (31.2%) compared to RPM**–**RPML (33.3%), RPL**–**RPML (32.8%), and RPML**–**RPML (33.1%). Nitrogen efficiency was affected (*p* < 0.001) by time, because N efficiency linearly decreased with the progression of lactation from 30 to 150 DIM, with the highest (*p* < 0.05) N efficiency at 30 DIM and the lower one at 150 DIM.

**Figure 9:**
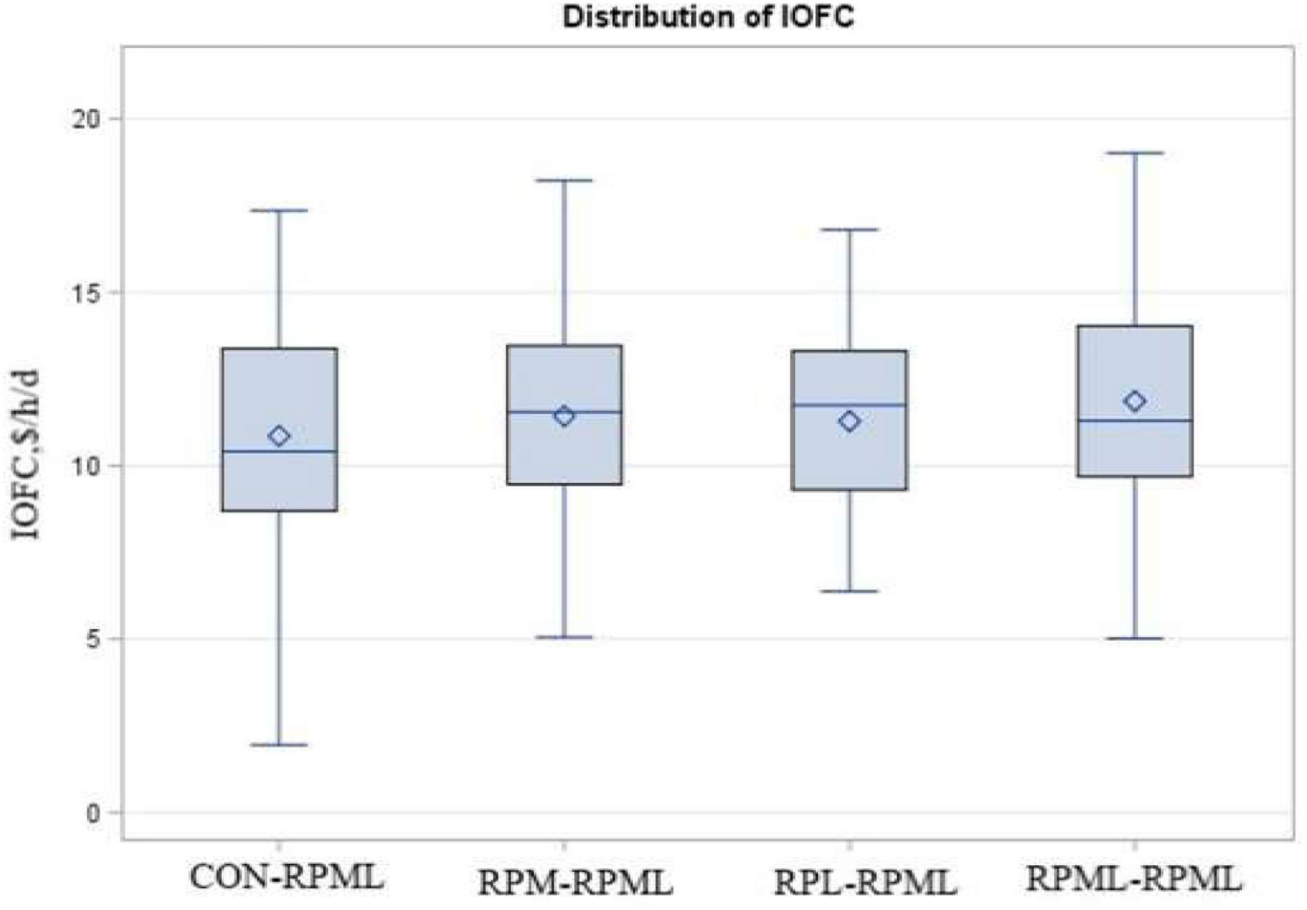
Effect of supply ruminally protected Met and Lys to transition and milking dairy cows on income over feed cost (IOFC). Values are means; standard errors are represented by vertical bars.

Income over feed cost tended (*p* = 0.10) to increase with AA supply (Table 5, Figure 9); as cows in the group fed RPML**–**RPML (11.86 $/h/day) had a higher (*p* < 0.05) IOFC than CON**–**RPML (10.86 $/h/day), and IOFC tended (*p* = 0.08) to increase in RPML**–**RPML (11.86 $/h/day) compared to RPM**–**RPML (11.42 $/h/day) and RPL**–**RPML (11.28 $/h/day). Time affected IOFC (*p* < 0.001), in which the highest (*p* < 0.05) IOFC (12.45 $/h/day), showed at 30 DIM compared to IOFC at 60 DIM (12.02 $/h/day), 90 DIM (11.27 $/h/day), 120 DIM (11.11 $/h/day), and 150 DIM (9.9 $/h/day), meaning that the IOFC decreases linearly (*p* < 0.05) with the progression of lactation from 30 to 150 DIM, when the cows cross over the peak of milk.

### Effect of Amino Acids Supply on Fertility

**Figure 10:**
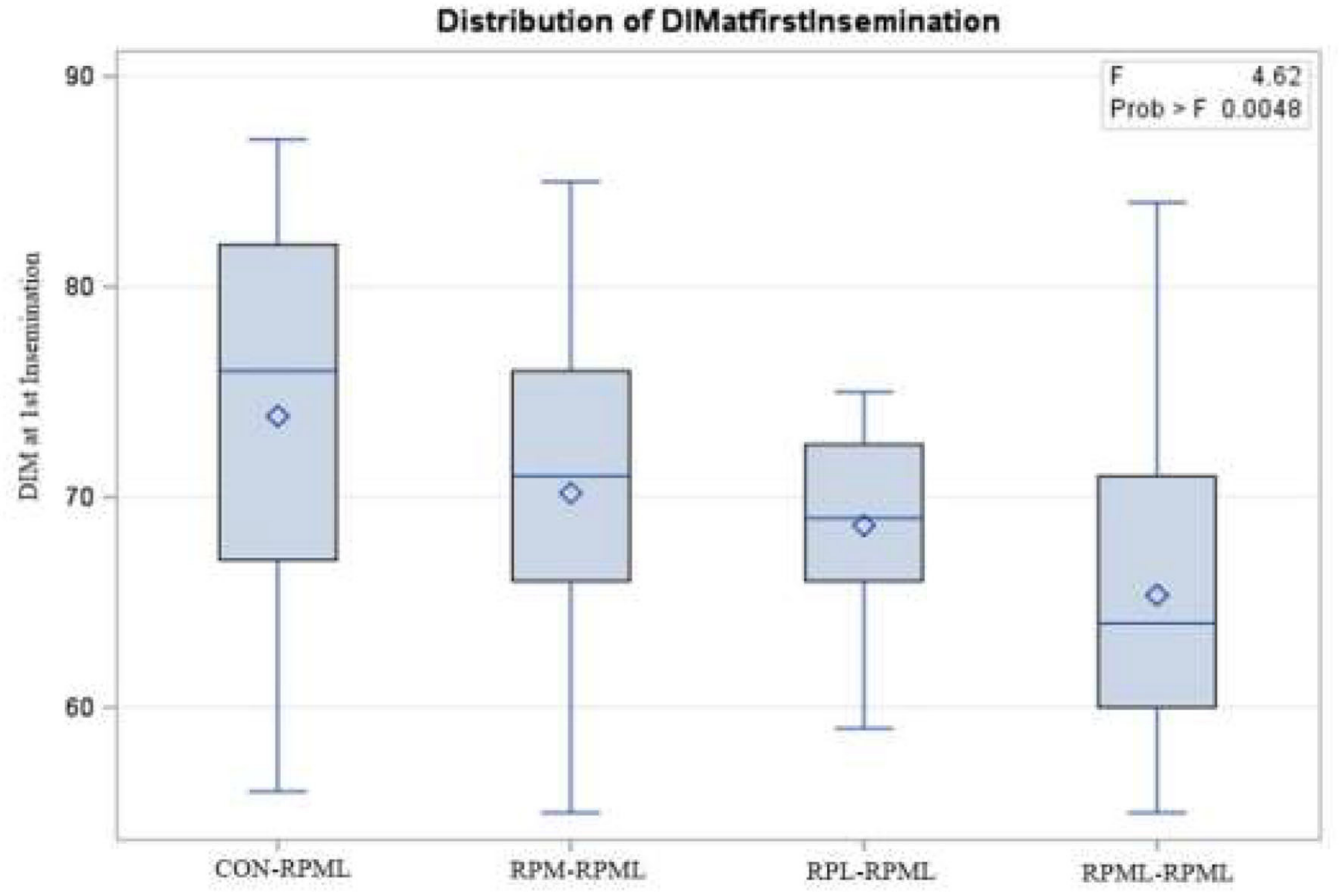
Effect of supply ruminally protected Met and Lys to transition and milking dairy cows on days to 1st insemination. Values are means; standard errors are represented by vertical bars.

**Figure 11:**
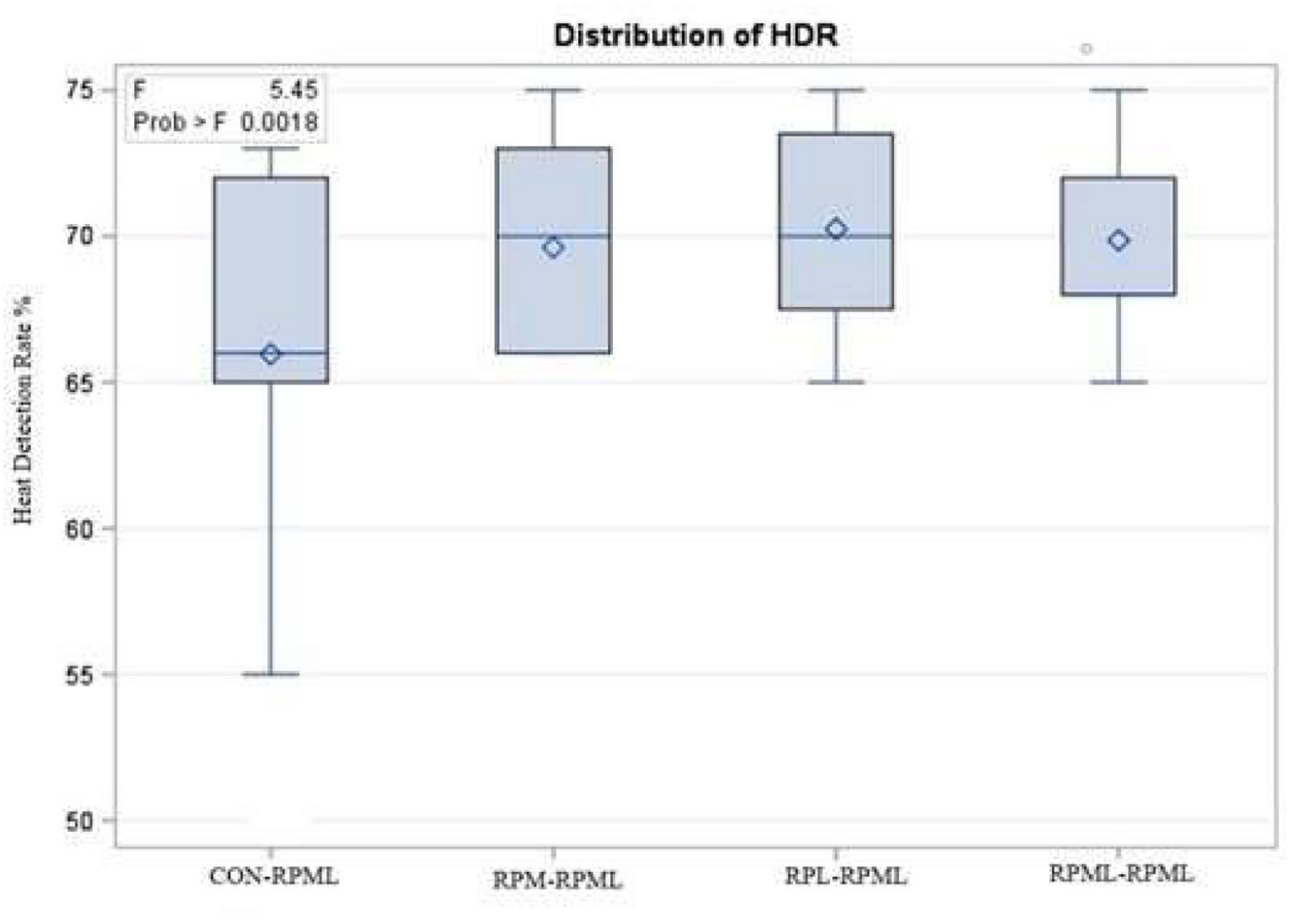
Effect of supply ruminally protected Met and Lys to transition and milking dairy cows on heat detection rate. Values are means; standard errors are represented by vertical bars.

**Figure 12:**
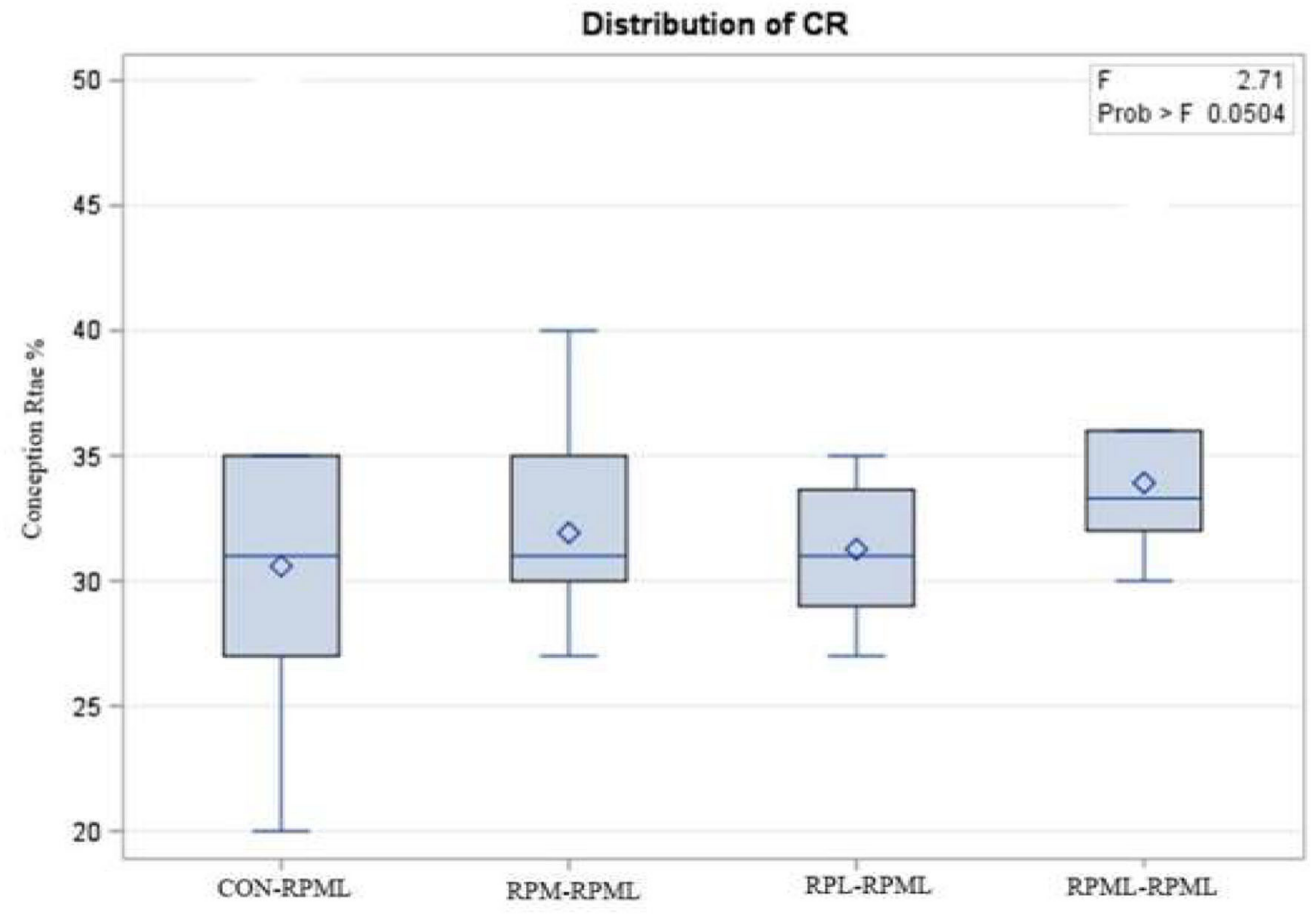
Effect of supply ruminally protected Met and Lys to transition and milking dairy cows on conception rate. Values are means; standard errors are represented by vertical bars.

**Figure 13:**
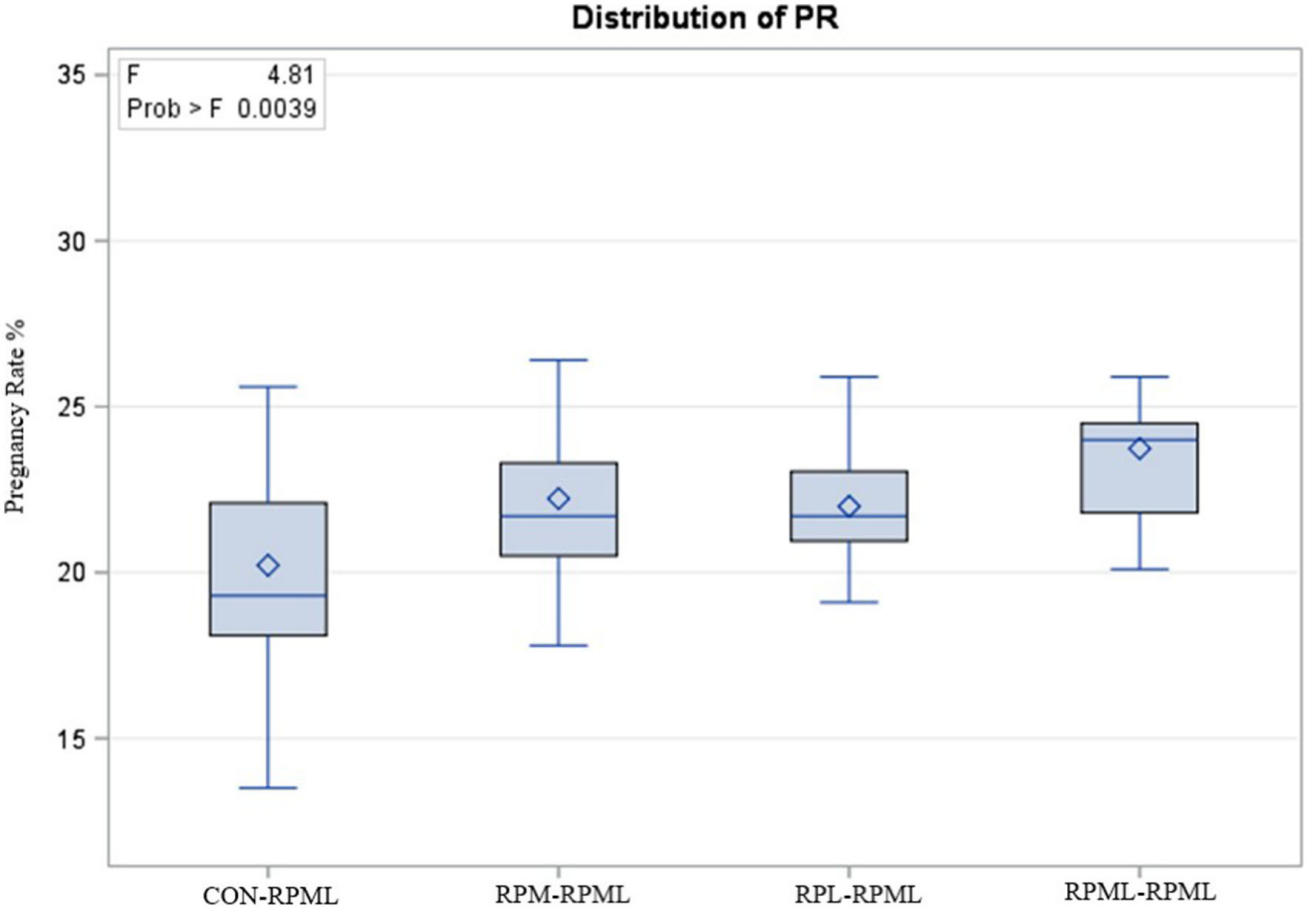
Effect of supply ruminally protected Met and Lys to transition and milking dairy cows on pregnancy rate. Values are means; standard errors are represented by vertical bars.

The effect of feeding RPL and RPM to transition dairy cows on fertility is presented in Table 6. There was a significant Trt effect on DIM at 1st insemination (*p* < 0.004), as cows in CON**–**RPML treatment spent long days to get inseminated (73.85 days) than other cows 70.19, 86.65, and 65.33 days for RPM**–**RPML and RPL**–**RPML and RPML**–**RPML, respectively (Table 6, Figure 10). There was no difference on DIM at 1st insemination between RPM**–**RPML and RPL**–**RPML and RPML**–**RPML (*p* > 0.10). Heat detection rate was lower in cows fed CON**–**RPML diet (*p* < 0.002) compared with those cows that were fed other diets (65.96 vs. 69.62, 70.25, and 69.85%), for CON**–**RPML, RPM**–**RPML, RPL**–**RPML, and RPML**–**RPML, respectively (Table 6, Figure 11). Conception rate was higher (*p* < 0.05) for cows in RPML**–**RPML group than other cows (Table 6, Figure 12). There was no difference in conception rate between CON**–**RPML, RPM**–**RPML, and RPL**–**RPML (*p* > 0.10). The pregnancy rate was affected by RPAA supplementation, in which transition cows that received RPAA had a higher pregnancy rate than those cows that fed an unsupplemented diet (*p* < 0.004; Table 6, Figure 13). Cows that consumed RPML**–**RPML (23.74%) had a higher (*p* = 0.04) pregnancy rate than CON**–**RPML (20.22%), RPM**–**RPML (22.23%), or RPL**–**RPML (22.0%). Pregnancy rate was similar (*p* = 0.81) between RPM**–**RPML and RPL**–**RPML.

**Table 6:** Feeding ruminally protected Met and Lys to transition dairy cows and its subsequent effect on fertility.

**Variable**	**Treatment** ^**1**^					***p*-value**
	**CON–RPML**	**RPM–RPML**	**RPL–RPML**	**RPML–RPML**	**SEM^**2**^**	**Trt^**3**^**
**Fertility parameters**						
Days in milk at 1^st^ insemination	73.85^a^	70.19^ab^	68.65^b^	65.33^b^	8.02	0.004
Heat detection rate %	65.96^b^	69.62^a^	70.25^a^	69.85^a^	4.25	0.002
Conception rate %	30.59^b^	31.91^ab^	31.27^b^	33.19^a^	4.14	0.050
Pregnancy rate %	20.22^c^	22.23^ab^	22.00^b^	23.74^a^	3.22	0.004

a,b,c *Mean values with different superscripts in the same row were significantly different (p < 0.05)*.

1 *High milking diets (from 22**–**150 days in milk) all cow diets of CON-RPML, RPM-RPML, RPL-RPML, and RPML-RPML, were provided with the combination of Met and Lys at a rate of (RPM; 0.17% DM& RPL; 0.41% DM, and NE_L_ = 1.80 Mcal/kg DM)*.

2 *Standard error means of all treatments*.

3 *Trt = effect of treatment*.

## Discussion

The important results from this study were as follows: (1) an increase in the peak of milk production and the less time to reach to peak with a supply combination of RPL and RPM, (2) increased milk protein percentage and ECM yield due to dietary RPL and RPM supply, with no effects on feed efficiency, (3) improve N efficiency with given RPL and RPM, and (4) increased pregnancy rate by providing RPL and RPM continual from transition till 150 DIM, and consequently improve the fertility efficiency.

### Effect of Amino Acids Supply on Dry Matter Intake

In the first stage of this study that focused on a continual supply of either RPL or RPM or two in combination during the transition period that lasted for 3 weeks before until 3 weeks after calving, we observed an increase in DMI in response to the supplies of RPL and RPM to transition dairy cows (18). The response of DMI to RPAA supply during the beyond period to the transition period is almost matched with that response that has previously been detected during the transition period (in which all cows received a combination of RPL and RPM). Stimulating DMI resulted in greater energy and total MP intake, which supported greater production and spared BW loss in this study, mainly to increase indispensable amino acids (IAA) efficiency.

Increased DMI was generally reported in response to the supply of RPL and RPM separately or in combination to dry and lactating cows (16, 42–45), consistent with the current findings as well as with our results during the transition period using the same cows that have continued in this study, but the response not universally (12, 27, 46–50). So far, the mechanism of action that governs the response of feed intake to amino acids supply in ruminants has not yet been completed. Nevertheless, the mechanism linking AA and DMI has been shown in rodents to affect food intake. There is considerable evidence for neural control of food intake in response to imbalanced AA diets in rats (51, 52), and this can also apply to ruminants in the same mechanism. Also, there is evidence that other factors are operating for cows deficient in multiple AA (53). However, the mechanisms underlying the control of the feeding response to the dietary model remain not fully understood (52), and more research is needed. This is the first study that evaluated the effects of feeding a combination of RPL and RPM on milk production coupled with the reproductive efficiency of lactating Holstein cows.

### Metabolizable Protein Balance and CNCPS Evaluation of Diets

In this study, the high MP balance observed for cows fed CON**–**RPML diet may be explained by the increase in MP intake (driven by the increase of DMI also, and at the same time dietary balance for AA profile assist in improving DMI) at a greater rate than AA secreted in the milk, thus making a higher MP balance compared to the other cows. However, a 524 g/day MP deficiency was detected for cows consumed the CON**–**RPML diet during the early lactation period (18), and this decrease is accompanied by a decrease in milk production and its components and a decrease in BCS also during the first 3 weeks of lactation compared to other treatments (18). The improvement in MP balance in CON-RPML beyond of transition period (when a combination of RPL and RPM was fed) resulted in an improved AA profile and increased milk production during the high milking period. Perhaps, the improvement in MP balance explains how the milk production increased in CON-RPML and became almost similar to RPM**–**RPML and RPL**–**RPML treatments during the high milking period.

### Effects of Amino Acids Supply on Body Condition Score

Body condition score improved in this study due to the high DMI supported by supplementing RPAA, which minimized BCS changes at 150 DIM compared with BCS at 21 DIM. At the same time, the cows with normal BCS during the post-calving period are still maintained at the level of the BCS. In this study, we detected that the increase in BCS during the high lactation period is mostly proportional to the BCS during the early lactation period; this means that cows with normal BCS during the early lactation period are better now (RPML**–**RPML). Additionally, the cows with the lowest BCS during the early post-calving period are the lowest BCS during the high milking period as well (CON**–**RPML). Dissimilarly, other studies indicated that providing RPM or RPL did not affect the BCS of dairy cows (12, 16, 43, 44, 54, 55); in most of these studies, but not all, DMI did not increase, or less increase, and perhaps, this explains the lack of response of BCS to RPAA supply, unlike the results of the current experiment. Body condition score is a dynamic indicator to determine the mobilization of fatty tissue from the body stores; therefore, BCS is a very important measure from a practical point of view as an indication for evaluating the level of nutrition and farm management efficiency in commercial dairy farms.

### Effect of Amino Acids Supply on Days to Peak and Peak of Milk

In this study, the higher peak of milk yield and shorter days-to-peak of milk could be explained by the success of the transition period as a result of the addition of RPAA, and a reasonable increase in DMI during both transition and early lactation period, as it is used as an indicator of nutritional management of transition cows. This is also connected with a reduction in the concentration of BHB in the blood in cows fed RPAA, which leads to improved animal health. All these factors combined lead to high milk production during the peak period and reaching the peak in a short period after calving for cows consumed RPAA during the transition period. The transition phase represents the time of greatest risk to the dairy cow. Metabolic disorders and physiological changes, combined with other factors, are the events that limit the cow in its ability to achieve higher peaks. The health events have both major nutritional influences and environmental and social causes and most often are intertwined. Most nutritional efforts have been focused on pre-and post-calving nutrition periods. Over the years, these improved feeding practices have been responsible for much of the increase in milk production in dairy farming.

The nutrition side of transition is much more complicated, with interactions between protein, minerals, vitamins, and energy status opening the door to metabolic challenges. This, combined with the effects of weather and farm management level, provides the most opportunity for things to go wrong at any time in the calendar of the cows. In this study, supplementing a combination of RPL and RPM may help to improve AA profile, which stimulates DMI, leading to greater EB and metabolizable protein intake, sparing BCS, consequently assisting minimize the occurrence of ketosis and helping the cow metabolize fat more efficiently during the transition and beyond period. In addition to the good farm management, all the above factors can allow cows to achieve better performance in early lactation, leading to a higher peak of milk and reaching the peak of milk in the shortest possible time in this study.

### Effect of Amino Acids Supply on β-Hydroxybutyrate Concentration

In this study, as we reported earlier during the transition period, Elsaadawy et al. (18) we showed that the lower BHB in response to the supply of a combination of RPL and RPM can be explained mainly by the greater consumption of DMI that is driven by balancing AA profile (18), in complement to other auxiliary factors. Excessive lipid mobilization results in incomplete hepatic oxidation and release of ketone bodies (56, 57), such as acetoacetate, BHB, and acetone. While, during the high milking period (from 22 to 150 DIM), all cows were fed a mixture of RPL and RPM and all become in positive energy and metabolizable protein balance, meaning that there is no mobilization for adipose tissue, this explains the normal level of BHB for all cows after 30 DIM (<1.2 mmol/L), in addition to increase DMI. During negative energy balance (around calving and up to a few weeks after calving), there is a large uptake of adipose tissue-derived long-chain fatty acid (FA) by the liver, which would have resulted in incomplete oxidation of non-esterified fatty acid (NEFA) and, consequently, elevated ketone bodies (56), which minimized with RPAA supply in this study.

Supplemental RPAA to dairy cows had varied effects on β-hydroxybutyrate concentration. Decreased BHB concentration had been reported when RPL and RPM were fed to dairy cows (27, 43, 58), but not in all cases (13). The difference in BHB concentration in response to RPAA supply is explained by many reasons, including the concentration of RPAA, length of the experimental period, type of animal (multiparous or primiparous), time of supplementation (e.g., close-up or post-calving, or both, or high milking, or mid-lactation cows), the composition of AA (e.g., RPAA, or analog, or AA derivatives), the methods of addition (e.g., mixed with TMR, or top-dressed), and the environmental condition as well as the farm management level. One or both of these factors may affect the animal's response and the concentration of BHB to AA supplementation.

The liver's free fatty acid oxidation rate appears to regulate feed intake in ruminants. Allen and Piantoni (59) reported that hepatic oxidation of NEFA stimulated brain feeding centers *via* hepatic vagal nerve to suppress intake. Based on this observation, it is probable that liver concentrations of FA or FA-derived metabolites such as BHB are being sensed rather than oxidation. In either case, exporting more FA as very low-density lipoprotein (VLDL) would lead to less oxidative stress in the liver and avoid feed intake suppression, this partially explains the reason for the low BHB concentrations in this experiment. Another reason to explain the lower BHB with AA supply suggests that RPM and RPL might have improved hepatic lipid metabolism and increased carnitine bioavailability. Osorio et al. (16) showed a tendency to decrease the occurrence of clinical ketosis when RPM was fed to transition cows, suggesting that supplied RPM might have improved hepatic lipid metabolism. Lipotropic agents such as Met or choline assist in lipid export from the liver by stimulating VLDL formation (56). Consequently, that response may lead to a decrease in hepatic triacylglycerol (TAG) accumulation and formation of ketone bodies (56, 60), which may also assist in the decreasing of BHB during the transition period and the later period by RPAA supply.

### Effects of Amino Acids Supply on Milk Production and Composition

In this study, matched with our first hypothesis, continual feeding of a combination of RPL and RPM throughout the transition and beyond (during the high milking period from 22 to 150 DIM) allows the cows to produce more milk protein yield and higher content, besides the increased milk production. Some EAAs are considered limiting for milk protein synthesis under specific dietary conditions in dairy cows, typically Met and Lys (17), and probably His. The increase in milk protein percentage and yield observed in this study in response to RPL and RPM agreed with what was previously obtained (9, 14, 16, 61, 62), but not always (27, 63, 64). Consistent with other studies, there was a positive response of dietary RPL and RPM supply on milk yield for transition and lactating and beef cows (13, 14, 16, 61, 65, 66), but this response is not universal (12, 27, 67–69).

In this study, milk lactose yield and protein increased with RPAA supply resulting in the use of a greater proportion of the unsupplemented IAA for milk protein. Junior et al. (61) demonstrated that improving AA absorption efficiency increases milk production in high-lactating dairy cows. Metabolizable protein supply has been recognized as the limiting factor for the absorption of AA in the mammary gland, which will be decided the yields of protein, lactose, and subsequently milk production (70, 71), as milk yield is a derivative from these components. Increasing the supply of MP would increase protein yield, milk production, and the amount required for lactose synthesis (13, 70), which was also observed in this study. Milk protein responses to Met are well documented, and responses to Lys and His are somewhat more changeable but have often been observed (72, 73). Besides Met, Lys, and His, it shows that the other two IAAs of leucine (Leu) and isoleucine (Ile) are also involved in driving milk protein production (74, 75).

The significant milk production response to RPAA in this study may be due to the increased efficiency of absorbed AA use (in addition to providing sufficient MP) that stimulates DMI and drive milk production. However, there are many reasons for the inconsistent in milk yield and constituents in response to RPAA supply; it is explained by the variability of protection methods for RPAA, which would make a difference in bioavailability, which may have accounted for the differences in results between studies; there were differences in the basal diets feedstuff, milk production level, and the metabolizable amount of Met and Lys g/MP. Additionally, the study length and animal type (multiparous, primiparous, pregnant, etc.), lactation phase, farm management level, and cow's well-being may also affect animal response to RPAA supply.

A deficiency in energy, excess dietary protein, and an imbalance between energy and protein could increase MUN (76, 77); this did not happen in this study. The lower MUN shown in this study indicates greater efficiency of N utilization. Lowered MUN has previously been observed by supplying RPAA form, including Met, Lys, leucine, threonine, and isoleucine separately or two in combination to lactating dairy cows (28, 29, 78), with no response in others (13, 27, 43, 61, 68). In this study, continual supplementing RPAA to transition and lactating dairy cows may improve udder health as indicated by the lowered milk SCC, as reported earlier in this study during the perinatal period (18, 29). Supply RPM plays a vital role in enhancing the immune status in dairy cows through enhanced phagocytosis and oxidative functions, which has been revealed when RPM was fed to dairy cows pre-calving and post-calving continuously (66), and it may apply for Lys as well. The current result of lowered SCC was consistent with other studies that fed RPM and RPL to pre-calving dairy cows (27), but this effect is not common (9, 13, 61, 78). Many reasons can cause an increase in milk SCC: one of these inflammation in the mammary gland tissues (79), cow productivity, health status, lactation, lactation phase, and animal breed (80).

### Effect of Amino Acids Supply on Nitrogen Efficiency and Income Over Feed Cost

The improvement in N efficiency in this study can be clarified by the increase in milk production, driven by the increase in DMI and dietary AA balance. Nitrogen efficiency is relatively low in dairy cows, with an average of 25%−35% of N feed intake secreted into N milk protein (81) and nearly all the residual N excreted into feces and urine with an average rate of 72% (82). A previous study showed that feeding of Met or Lys or both could increase N efficiency (83), similar to the results from this study. However, in the period of the peak of milk (38 ± 15 DIM), RPL and RPM supply (5 g/day Met and 10 g/day Lys, intestinally absorbed) observed a decrease in N efficiency in Holstein dairy cows (84); this may be due to the small amount of absorbed RPL and RPM that were consumed or the lack of control of AA balance in that study. Other authors reported no improvement in N efficiency when they evaluated Met supplementation in a digestibility experiment (85).

Income over feed cost was increased for cows that consumed RPML-RPML in this study due to the greater ECM production. The authors mentioned IOFC here to light the importance of RPAA supply from an economic point of view. For example, the IOFC from the cows fed RPML-RPML would be $1.0 more than that from CON-RPML cows. It could improve many aspects of production efficiency besides the economic profit. So, the IOFC is a gross margin concept.

### Effect of Amino Acids Supply on Fertility and Reproductive Parameters

The mechanisms that underlie the increased pregnancy rate by feeding RPL and RPM in multiparous Holstein cows remain unclear. The effects of Met could be at different stages of embryonic development and are linked to multiple biochemical pathways caused by insufficient Met, such as an overall depression in embryonic protein production, decreased the activity of one-carbon (1-C) metabolism, probably leading to a reduction of DNA methylation, or reduction in embryonic polyamines. Toledo et al. (9) indicated that multiparous cows might be more prone to Met deficiency than primiparous cows due to increased milk protein yield and milk production and thus could lead to an increased need for Met; this is one of the main reasons for using multiparous cows in the present experiment. Toledo et al. (9) also reported that the Met circulating rate was lower in multiparous cows compared to primiparous cows (9). Thus, insufficient circulating Met concentrations in multiparous cows may limit intrauterine Met concentrations, leading to a delay in embryonic development. A recent study has shown that multiparous cows have a larger uterine size and lower fertility than primiparous cows (86). There is a large increase in concentrations of AA in the uterine histotroph during early pregnancy in sheep (8) and cattle (7), including a large increase in Met, Lys, and other EAA as well, and this may be important for optimal embryonic development. Toledo et al. (9) showed that cows with a larger uterine volume may have reduced AA concentrations in the uterine histotroph and probably larger AA requirements during the elongation and early placentation period of pregnancy. Besides, an *in vivo* study reported that providing RPM to lactating dairy cattle rations created dramatic modulation in gene expression in embryos, in general decreasing the concentrations of mRNA in early embryos (87).

Feeding a combination of RPL and RPM in this study may induce changes in the early embryo that are consequently visible in the later phases of pregnancy. Souza et al. (88) demonstrated that Met consumption did not impact fertilization and gross morphological quality of day 7 embryos, but the diet supplied with Met altered embryonic gene expression, particularly leading to reducing the expression of many specific genes correlated to fetus development (e.g., VIM, BCL2A1, IFI6, and TBX15) and immune response (e.g., LCP1, NKG7, TYROBP, SLAMF7, and BLA-DQB). Recently, it has been proven that arginine has an important role on pregnancy in the early stages. Sun et al. (89) reported that maternal rumen-protected arginine supply to pregnant ewes fed a restricted diet (50% of the requirement between days 35 and 110 of pregnancy) improving the endocrine metabolic homeostasis in the fetus and increasing the availability of AA in the embryonic liver and longissimus dorsi muscle as well as its effect on the expression of somatotropic axis genes, and it is possible that this mechanism applies to Lys and thus helps us in understanding the mechanism of Lys in dairy cows. An *in vitro* study (22) indicated a surprisingly low Met requirement (7–21 μM) for developing morphologically normal bovine embryos. However, treatment of *in vitro*-produced bovine embryos with ethionine (a Met anti-metabolite) reduced embryo development at the blastocyst stage, and the addition of S-adenosylmethionine (SAM) could partially restore embryonic development in the presence of methionine (90).

There may be a need for supplementing an appropriate amount of Met to the cows' diets to ensure a proper embryo development because of the effects on DNA methylation, fetal gene expression, general protein synthesis pathways, and specific metabolic pathways, such as polyamines, which may be pivotal for optimal progression of the pregnancy and Lys as well. Lately, an *in vivo* study demonstrated that cows received RPM-generated early embryos with lower DNA methylation but higher lipid content proposing that Met supply may affect energy metabolism and, consequently, embryo survival (23). Moreover, in other species, Met metabolism participates in synthesizing polyamines (91, 92). The reduction of polyamines is generally correlated with the weakness of early embryo development, attachment, growth of extra-embryonic structures and placenta, and steroidogenesis (25, 93); these highlight other possible pathways that may make pregnancy loss in animals with deficient Met, and this may be one of the reasons to explain the improvement of pregnancy in this study as a result of feeding the Met in addition to Lys as well.

Fertility efficiency has been improved in response to dietary supply of RPAA, either RPL or RPM or two in combination with dairy cows (9, 19), or small or even no effects (20, 21). Toledo et al. (9) indicated that RPM supply (21.2 g/h/day; to be within 2.34% of MP) from 30 until 126 DIM to multiparous lactating cows decreased pregnancy loss between days 32 to 61 of pregnancy. Ardalan et al. (19) reported that dairy cows that consumed RPM (18 g/day) from 4 weeks pre-calving through 20 weeks post-calving improved conception rate and consequently decreased open days, in line with the results obtained from this current experiment. Additionally, studies in sheep (24, 25) and dairy cattle (7, 26) have reported that Met was concentrated in uterine and embryonic fluids, emphasizing the role of increased uterine Met in normal embryonic development and survival, and this partly explains the improvement of fertility in this study in dairy cows as a result of supplementing RPAA during the transition period and later period. Feeding dairy cows a low protein diet supplied with Met (14% CP, 40 g/day Met) from parturition to 120 DIM had a less effect on reproductivity, as 1st conception rate was 28.5 and days open was 134 days (94), which was lower values than what was obtained in this study by providing RPL and RPM. In contrast, Polan et al. (21) reported that supply RPM and RPL had no effects on DIM at 1st insemination, service per conception, and calving interval in Holstein dairy cows.

## Conclusions

In this study, continual consumption of a combination of RPL and RPM throughout the transition and high milking period increased DMI, ECM, and milk composition production, with no effects on feed efficiency. There was an improvement in N utilization as well as enhanced fertility efficiency in response to AA supply.

## Data Availability Statement

The raw data supporting the conclusions of this article will be made available by the authors, without undue reservation.

## Ethics Statement

All procedures were approved by the Animal Care and Use Committee of the Institute of Animal Science, Chinese Academy of Agricultural Sciences, Beijing, China (no. IAS20180115).

## Author Contributions

SE: conceptualization, conducted the experiment, laboratory analysis, writing—original draft, and formal analysis. ZW: investigation. DB: supervision, project administration, and funding acquisition.

## Funding

This research was partially supported by the Key Research and Development Program of the Ningxia Hui Autonomous Region (2021BEF02018), the International Atomic Energy Agency Technical Co-operation and Assistance Programme (no. CPR5025), the Agriculture Science and Technology Innovation Program (ASTIP-IAS07-1), Chinese Academy of Agricultural Science and Technology Innovation Project (CAAS-XTCX2016011-01), and Beijing Dairy Industry Innovation Team (BAIC06-2022).

## Conflict of Interest

The authors declare that the research was conducted in the absence of any commercial or financial relationships that could be construed as a potential conflict of interest.

## Publisher's Note

All claims expressed in this article are solely those of the authors and do not necessarily represent those of their affiliated organizations, or those of the publisher, the editors and the reviewers. Any product that may be evaluated in this article, or claim that may be made by its manufacturer, is not guaranteed or endorsed by the publisher.
